# Baricitinib as monotherapy and with topical corticosteroids in moderate-to-severe atopic dermatitis: a systematic review and meta-analysis of dose-response

**DOI:** 10.3389/falgy.2024.1486271

**Published:** 2024-11-14

**Authors:** Ibrahim H. I. Almoghayer, Abdul Mateen Soomro, Shah Dev, Muskan Turesh, Ateesh Kumar, Ravi Kumar, Aashish Meghjiani, Syeda Lamiya Mir, Muhammad Hassaan, Rehan Qureshi, Vishal Kumar, Taimoor Ashraf, F. N. U. Deepak, Mohammad Arham Siddiq, Abdul Haseeb, Ayush Kumar

**Affiliations:** ^1^ Liaquat University of Medical & Health Sciences, Jamshoro, Pakistan; ^2^ Department of Dermatology, Avicenna Tajik State Medical University, Dushanbe, Tajikistan; ^3^ Department of Dermatology, Ghulam Muhammad Mahar Medical College, Sukkur, Pakistan; ^4^ Dow Medical College, Dow University of Health Sciences, Karachi, Pakistan; ^5^ Department of Dermatology, Nishtar Medical College, Multan, Pakistan; ^6^ Department of Dermatology, Shaheed Mohtarma Benazir Bhutto Medical College Lyari, Karachi, Pakistan; ^7^ Department of Dermatology, Jinnah Sindh Medical University, Karachi, Pakistan; ^8^ Department of Dermatology, Vayodha Hospitals, Kathmandu, Nepal

**Keywords:** atopic dermatitis, baricitinib, JAK inhibitors, eczema area and severity index, treatment-emergent adverse events

## Abstract

**Introduction:**

Atopic dermatitis (AD) is a chronic inflammatory skin disorder that affects millions worldwide, presenting challenges in managing symptoms and quality of life. Current treatments include topical corticosteroids (TCS), but novel approaches, such as Janus kinase (JAK) inhibitors, show promise. Baricitinib, a selective JAK1 and JAK2 inhibitor, targets cytokines involved in AD and offers potential benefits beyond traditional therapies.

**Methods:**

A systematic review and meta-analysis of randomized controlled trials (RCTs) was performed to evaluate the efficacy and safety of baricitinib in treating moderate-to-severe AD. We followed PRISMA guidelines and assessed data from PubMed, Cochrane Central, ScienceDirect, and ClinicalTrials.gov up to August 2024. The analysis included trials comparing baricitinib to placebo, with or without TCS, evaluating outcomes such as Investigator's Global Assessment (IGA) scores, Eczema Area and Severity Index (EASI) scores, and safety profiles.

**Results:**

Six RCTs involving 2,595 participants met the inclusion criteria. Baricitinib demonstrated significant improvements in IGA scores, EASI scores, Dermatology Life Quality Index (DLQI), and other outcome measures compared to placebo. The efficacy was consistent across different dosages (1 mg, 2 mg, 4 mg) and whether baricitinib was used with or without TCS. Safety analyses revealed a significant increase in treatment-emergent adverse events (TEAEs), particularly with the 2 mg and 4 mg dosages and with TCS.

**Conclusion:**

Baricitinib, both alone and in combination with TCS, significantly improves symptoms and quality of life in patients with moderate-to-severe AD, with efficacy consistent across dosages. The safety profile is overall acceptable, though a significant increase in TEAEs was observed, particularly with higher dosages and when used with TCS. Ongoing monitoring of TEAEs is recommended, and future trials with longer follow-up periods are suggested to better understand long-term outcomes.

## Introduction

Baricitinib, a selective JAK1/JAK2 inhibitor, blocks these cytokine pathways, targeting IL-4, IL-5, IL-13, and IL-31, which are key players in AD. Additionally, it inhibits Th1 and Th17 differentiation and reduces type-I IFN production, offering a promising therapeutic approach to modulate both innate and adaptive immunity in AD and other immune-mediated diseases.

Atopic dermatitis (AD), a widespread chronic inflammatory skin condition, is marked by a relapsing–remitting course, recurrent xerosis, eczematous lesions, and intense pruritus. Although AD can manifest at any age, it most commonly begins in infancy or early childhood and affects more than 200 million people worldwide, including up to 20% of children and 10% of adults (1, 2). A primary symptom of AD is pruritus, which initiates a vicious cycle of skin deterioration, itching, and irritation (3). The pathophysiology of AD is multifaceted and involves decreased function of the epidermal barrier, immunological dysregulation driven by Th2, hyperresponsiveness of the skin to inflammatory stimuli, and a feedback loop between inflammation and barrier dysfunction. Both environmental and genetic factors also play a role; genetic predisposition frequently marks the onset of related atopic disorders including allergic rhinoconjunctivitis and bronchial asthma (4).

AD is one of the chronic, incurable skin diseases that imposes significant financial, social, and psychological costs, along with systemic comorbidities. Psychologically, AD can lead to behavioral problems in children and an increase in psychiatric disorders and anxiety in adults, often coexisting with psychological distress such as depression and sleep disturbances, which drastically lowers quality of life. Socially, it affects family relationships, peer stigmatization, and interpersonal interactions. Additionally, the condition results in poor sleep, financial difficulties, and lifestyle changes that adversely impact family quality of life. Both direct costs (such as medical visits and treatments) and indirect costs (including lost productivity and work time) are substantial (5).

The therapeutic approach for AD seeks to mitigate symptoms and secure enduring disease management. Emollients and topical corticosteroids (TCS) represent the primary interventions, crucial for addressing mild to moderate AD and mitigating flare-ups. Topical calcineurin inhibitors, non-steroidal compounds that obstruct calcineurin-mediated T-cell activation, can be utilized as alternative or adjunctive treatments, particularly when concerns about steroid-induced atrophy arise (6). For individuals with inadequate responses to TCS or moderate-to-severe AD, phototherapy or systemic agents may be necessary. However, these treatments raise safety concerns for long-term use and have limited efficacy (7).

The Janus kinase (JAK) and signal transducer and activator of transcription (STAT) pathway plays a crucial role in the development and maintenance of AD, contributing to both acute and chronic phases of the disease. Cytokines like IL-4, IL-13, and IL-31 signal through JAK1, promoting Th2-mediated inflammation, which leads to pruritus, skin barrier dysfunction, and immune dysregulation. JAK2 is involved in signaling through IL-5 and IFN-γ, further driving eosinophil activation and chronic inflammation (8). Targeting this pathway offers a promising therapeutic strategy for attenuating the activation of various proinflammatory mediators involved in AD. JAK inhibitors are becoming more and more viable treatment options for this illness (9). Baricitinib, a selective JAK1/JAK2 inhibitor, blocks these cytokine pathways, targeting IL-4, IL-5, IL-13, and IL-31, which are key players in AD. Additionally, it inhibits Th1 and Th17 differentiation and reduces type-I IFN production, offering a promising therapeutic approach to modulate both innate and adaptive immunity in AD and other immune-mediated diseases (10, 11).

The efficacy and favorable safety profile of baricitinib for AD at different dosages, both with and without the addition of TCS, have been demonstrated in numerous randomized controlled trials (RCTs). To thoroughly assess the safety and efficacy of various baricitinib dosages, either alone or in combination with TCS, we conducted a systematic review and meta-analysis of RCTs. This analysis aims to provide robust evidence to guide clinical decision-making regarding the optimal dosage of baricitinib, with or without TCS, in the treatment of moderate-to-severe AD.

## Methods

This meta-analysis adhered to PRISMA guidelines for systematic reviews and meta-analyses and was conducted in alignment with the Cochrane Collaboration framework (12, 13).

### Literature search

A thorough literature search was conducted across PubMed, Cochrane Central, ScienceDirect, and ClinicalTrials.gov databases, encompassing all records from their inception through August 2024. This search was unrestricted by time, language, or sample size. The strategy employed Medical Subject Headings (MeSH) and keywords such as “atopic dermatitis,” OR “atopic eczema,” OR “eczema,” OR “allergic dermatitis,” combined with “baricitinib,” OR “LY3009104,” OR “JAK1/JAK2 inhibitor.” All the recognized research's study titles, abstracts, full texts, and bibliographies were carefully examined. Furthermore, references from relevant literature were carefully examined to find pertinent studies, without regard to publication language, geographic region, or ethnicity.

### Data extraction

Following the methodical search, hundreds of articles were located and added to EndNote Reference Manager (Version X7.5; Clarivate Analytics, Philadelphia, Pennsylvania). Within EndNote, duplicates were carefully identified and removed. Two reviewers independently assessed the titles and abstracts of the publications that met the inclusion criteria after carefully going over the whole contents. Following that, data was extracted from the trials that qualified and organized in an information-extraction table. Among the crucial data acquired were the first author, publication year, NCT number, sample size, participant age and sex, baseline characteristics, and duration of follow-up. Arguments about the choice of articles or data extraction were resolved by discussion or by consulting a third reviewer.

### Inclusion criteria and outcomes

#### Heading

The research study complied with the strict eligibility requirements for studies, which comprised the following: (a) studies offering pertinent outcome data; (b) adult patients (≥18 years) diagnosed with moderate-to-severe AD; and (c) RCTs with at least one intervention group receiving Baricitinib compared to a control group receiving a placebo. Accepted studies included oral dosages of 1, 2, or 4 mg of baricitinib given once day, either with or without TCS. Studies that used animal models, had unsuitable designs (such as non-randomized trials), lacked pertinent data, or were case reports, editorials, reviews, conference abstracts, or duplicate publications were among the many reasons they were eliminated.

### Outcomes of interest

At 16 weeks, the primary effectiveness outcome of interest was the proportion of patients treated with baricitinib who achieved an Investigator's Global Assessment (IGA) score of 0 or 1, on a scale ranging from 0 (clear skin) to 4 (severe disease). Secondary endpoints included the proportion of patients achieving 50%, 75%, and 90% improvement in the Eczema Area and Severity Index (EASI 50, 75, and 90), changes from baseline in the Dermatology Life Quality Index (DLQI), the percentage of participants achieving SCORing AD 75 and 90 (SCORAD 75, 90), the proportion of participants improving by four points on the Itch Numeric Rating Scale (NRS), the proportion of participants developing skin infections requiring antibiotic treatment, changes from baseline in Skin Pain NRS, changes in Body Surface Area (BSA) affected from baseline, and changes in the total score of the Patient-Oriented Eczema Measure (POEM) from baseline. The frequency of treatment-emergent adverse events (TEAEs) was used to evaluate safety.

### Risk of bias assessment

Using the RoB 2 methodology, every included RCT's risk of bias was carefully evaluated. This tool assesses several categories, such as the creation of random sequences, the concealment of allocations, participant and staff blinding, outcome assessment blinding, insufficient outcome data, selective reporting, and other possible sources of bias. The risk of bias in each category was carefully categorized as low, high, or unclear (14).

### Statistical analysis

Review Manager 5.3 (RevMan 5.3) was the software used for all statistical analyses. Applying the Mantel-Haenszel method to dichotomous outcomes yielded results that were represented as 95% confidence intervals (CIs) and risk ratios (RRs). The mean difference (MD) and associated 95% confidence interval (CI) for continuous variables were determined using the inverse variance approach. To take potential study heterogeneity into consideration, a random effects model was used. Cochrane's Higgins *I*² and Q statistics were used to assess heterogeneity. The I2 statistic calculates the proportion of variation between studies that is attributable to heterogeneity as opposed to chance; values less than 50% signify low heterogeneity, values greater than 50% indicate moderate heterogeneity, and values greater than 75% signify substantial heterogeneity (15). Subgroup analyses were carried out according to the dosage of baricitinib that was administered whether baricitinib was used with or without TCS. Sensitivity studies were designed to locate and address causes of significant heterogeneity using the leave-one-out method. When determining statistical significance, a strict *P* value criterion of less than 0.05 was used.

## Results

### Study screening and selection

**Figure 1:**
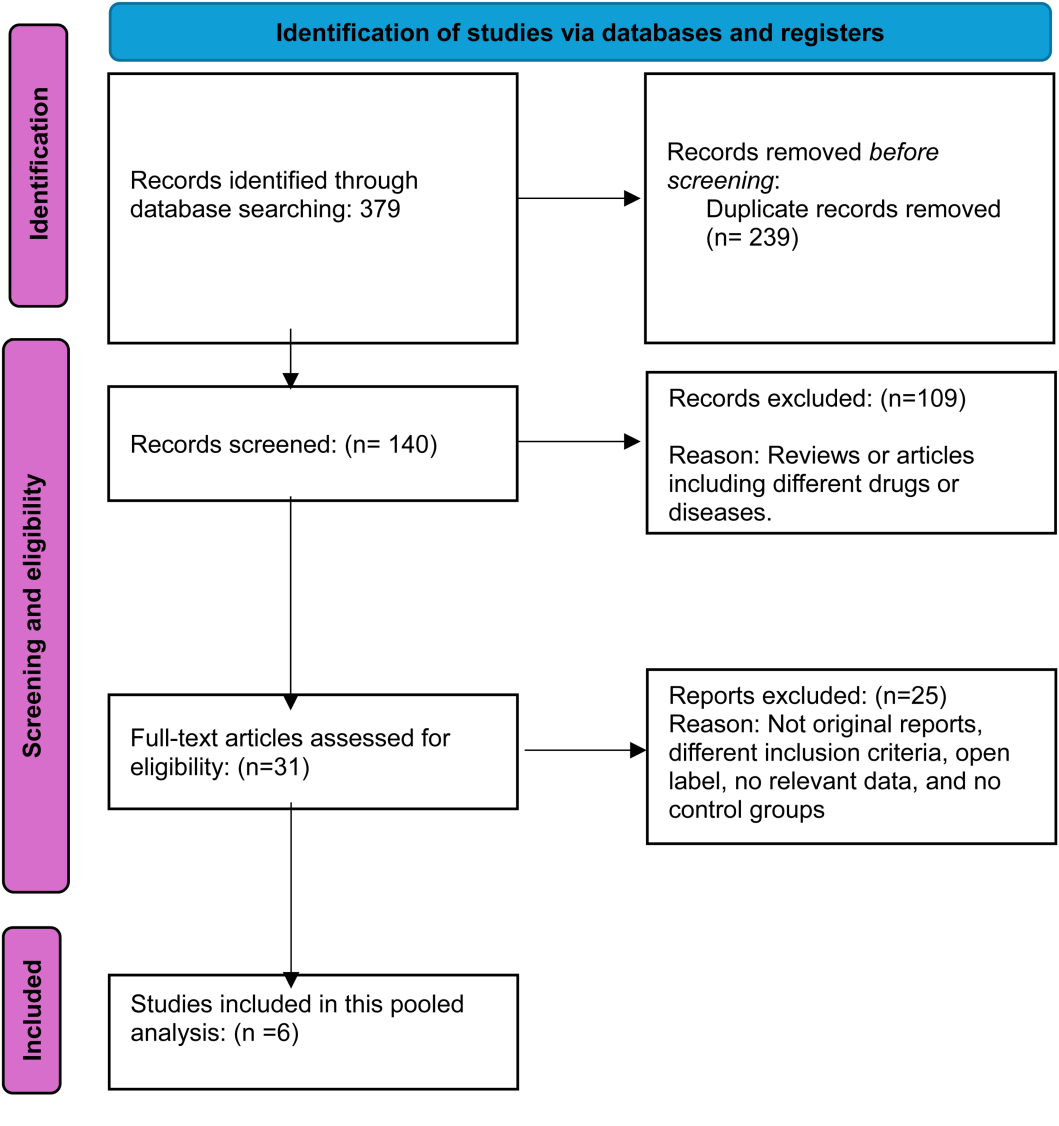
Prisma flow diagram.

The initial database search yielded 379 results, prompting a systematic review to eliminate redundancies. After removing 239 duplicates, 140 studies remained, and their titles and abstracts were meticulously screened. This rigorous process excluded 109 citations that were irrelevant to the research focus. Subsequently, the full texts of 31 studies were thoroughly examined for data on the intervention's safety and efficacy, leading to the exclusion of 25 articles that did not meet the inclusion criteria. The final analysis incorporated six studies comparing different dosages of baricitinib with placebo for AD, offering significant insights into the research topic. Figure 1 illustrates the PRISMA flow diagram, detailing the study selection process.

### Baseline and study characteristics

This analysis included six RCTs with a total of 2,595 participants. Of these, 1,704 patients received once-daily oral baricitinib, while 891 were assigned to the placebo group. The average age of participants ranged from 33 to 40 years, and the mean duration of AD among patients ranged from 19.7 to 28.4 years. The participant pool consisted of 1,577 men and 1,018 women. Table 1 details the baseline characteristics and study features of the included trials.

**Table 1:** Study and baseline characteristics.

Study name, year and NCT	Phase	Intervention	Total population	Age (years)	Sex (M/F)	Duration since AD diagnosis	IGA score of 4	EASI score	POEM	DLQI	BSA affected	SCORAD	Itch NRS	Skin Pain NRS	Outcomes	Follow up
				Mean (SD)	*N*	Mean (SD)	*N*	Mean (SD)	Mean (SD)	Mean (SD)	Mean (SD)	Mean (SD)	Mean (SD)	Mean (SD)		
Simpson EL et al. (AD5) 2021 NCT03435081	Phase III	Placebo	147	39 (17)	80/67	23 (17)	61	27.0 (11)	–	15 (7)	41.5 (23)	–	7.0 (2.4)	6.5 (2.7)	EASI 50, EASI 75, EASI 90, IGA score of 0 or 1, Change in DLQI, SCORAD 90, SCORAD 75, Itch NRS, TEAEs, Skin infection requiring antibiotic treatment, Skin Pain NRS, BSA affected, and POEM.	16 weeks
		Baricitinib 1 mg	147	40 (17)	75/72	24 (17)	62	27.7 (12)	–	15 (7)	41.4 (23)	–	7.2 (2.0)	6.5 (2.6)		
		Baricitinib 2 mg	146	40 (15)	69/77	24 (16)	61	26.6 (11)	–	15 (8)	39.7 (22)	–	7.3 (2.1)	6.7 (2.6)		
Reich K et al. (AD7) 2020 NCT03733301	Phase III	Placebo	109	33.7 (13.2)	71/38	22.0 (12.2)	48	28.5 (12.3)	20.9 (6.7)	15.0 (7.9)	48.1 (24.4)	66.6 (13.8)	7.4 (1.7)	6.8 (2.3)	EASI 50, EASI 75, EASI 90, IGA score of 0 or 1, Change in DLQI, SCORAD 90, SCORAD 75, Itch NRS, TEAEs, Skin infection requiring antibiotic treatment, Skin Pain NRS, BSA affected, and POEM.	16 weeks
		Baricitinib 2 mg	109	33.8 (12.8)	70/39	24.6 (14.8)	50	29.3 (11.9)	21.0 (6.3)	15.0 (7.7)	50.6 (21.6)	66.8 (14.0)	7.0 (2.1)	6.3 (2.5)		
		Baricitinib 4 mg	111	33.9 (11.4)	75/36	25.5 (13.2)	50	30.9 (12.6)	21.4 (6.0)	14.7 (7.9)	52.1 (23.3)	68.3 (13.2)	7.0 (2.0)	6.0 (2.5)		
Guttman-Yassky E 2018 NCT02576938	Phase II	Placebo	49	37 (14.81)	24/25	18.16 (16.44)	–	21.8 (9.4)	20 (4.44)	14.6 (6.66)	–	54.56 (14)	7 (1.5)	–	EASI 50, EASI 75, EASI 90, IGA score of 0 or 1, Change in DLQI, and TEAEs.	16 weeks
		Baricitinib 2 mg	37	40 (19.25)	22/15	28.4 (16.44)	–	23.7 (11.5)	18 (9.6)	11.3 (7.4)	–	54.76 (8.29)	6.33 (2.22)	–		
		Baricitinib 4 mg	38	35.5 (16.29)	22/16	19.7 (18)	–	19.7 (9)	19 (11.1)	11.66 (6.66)	–	57.33 (11.4)	6.16 (2.96)	–		
Simpson EL et al. (AD1) NCT03334396	Phase III	Placebo	249	35 (12.6)	148/101	26 (15.5)	105	32 (13.0)	21 (5.6)	14 (7.4)	53 (23.1)	68 (14.0)	6.7 (2.0)	61 (25)	EASI 50, EASI 75, EASI 90, IGA score of 0 or 1, Change in DLQI, SCORAD 90, SCORAD 75, Itch NRS, TEAEs, Skin infection requiring antibiotic treatment, Skin Pain NRS, BSA affected, and POEM.	16 weeks
		Baricitinib 1 mg	127	36 (12.4)	78/49	27 (14.9)	53	29 (11.8)	20 (5.6)	13 (6.8)	47 (21.2)	66 (14.4)	6.1 (2.1)	5.5 (2.4)		
		Baricitinib 2 mg	123	35 (13.7)	82/41	25 (14.6)	52	31 (11.7)	21 (5.6)	13 (7.7)	50 (22.1)	68 (13.0)	6.4 (2.2	5.7 (2.6)		
		Baricitinib 4 mg	125	37 (12.9)	83/42	25 (14.9)	51	32 (12.7)	21 (5.6)	14 (7.1)	52 (21.8)	68 (12.9)	6.5 (2.0)	5.7 (2.4)		
Simpson EL et al. (AD2) NCT03334422	Phase III	Placebo	244	35 (13.0)	154/90	25 (13.9)	121	33 (12.8)	21 (6.3)	15 (8.1)	52 (21.7)	68 (12.7)	6.8 (2.2)	6.2 (2.5)	EASI 50, EASI 75, EASI 90, IGA score of 0 or 1, Change in DLQI, SCORAD 90, SCORAD 75, Itch NRS, TEAEs, Skin infection requiring antibiotic treatment, Skin Pain NRS, BSA affected, and POEM.	16 weeks
		Baricitinib 1 mg	125	33 (10.0)	80/45	24 (12.7)	63	33 (12.7)	20 (6.5)	15 (8.1)	55 (21.9)	67 (12.9)	6.4 (2.2)	5.7 (2.7)		
		Baricitinib 2 mg	123	36 (13.2)	65/58	24 (13.8)	62	35 (16.0)	21 (6.0)	14 (7.7)	55 (26.1)	69 (13.3)	6.6 (2.2)	6.2 (2.5)		
		Baricitinib 4 mg	123	34 (14.1)	82/41	23 (14.8)	63	33 (12.7)	20 (6.3)	14 (8.4)	54 (21.5)	68 (13.6)	6.6 (2.2)	6.0 (2.6)		
NCT03428100 (AD4)	Phase III	placebo	93	38.7 (13.6)	49/44	–	–	–	–	–	–	–	–	–	EASI 50, EASI 75, EASI 90, IGA score of 0 or 1, Change in DLQI, SCORAD 90, SCORAD 75, Itch NRS, TEAEs, Skin infection requiring antibiotic treatment, Skin Pain NRS, BSA affected, and POEM.	16 weeks
		Baricitinib 1 mg	93	38.9 (14.0)	58/35	–	–	–	–	–	–	–	–	–		
		Baricitinib 2 mg	185	37.3 (13.6)	133/52	–	–	–	–	–	–	–	–	–		
		Baricitinib 4 mg	92	38.7 (13.3)	57/35	–	–	–	–	–	–	–	–	–		

The six RCTs analyzed comprised one phase 2 study (16) and four phase 3 trials, conducted between 2019 and 2021, with results published across 3 studies (17–19), and an additional unpublished trial releasing results in 2021 (20). Three RCTs tested four treatment arms: baricitinib at 4 mg, 2 mg, 1 mg, and placebo. Two trials involved three treatment arms: baricitinib at 4 mg, 2 mg, and placebo, while one trial compared baricitinib at 2 mg, 1 mg, and placebo. In half of the trials, patients were treated with a combination of baricitinib and topical corticosteroids, while the remaining trials focused exclusively on baricitinib monotherapy. Outcomes were assessed uniformly at the 16-week mark.

### Assessment of quality

**Figure 2:**
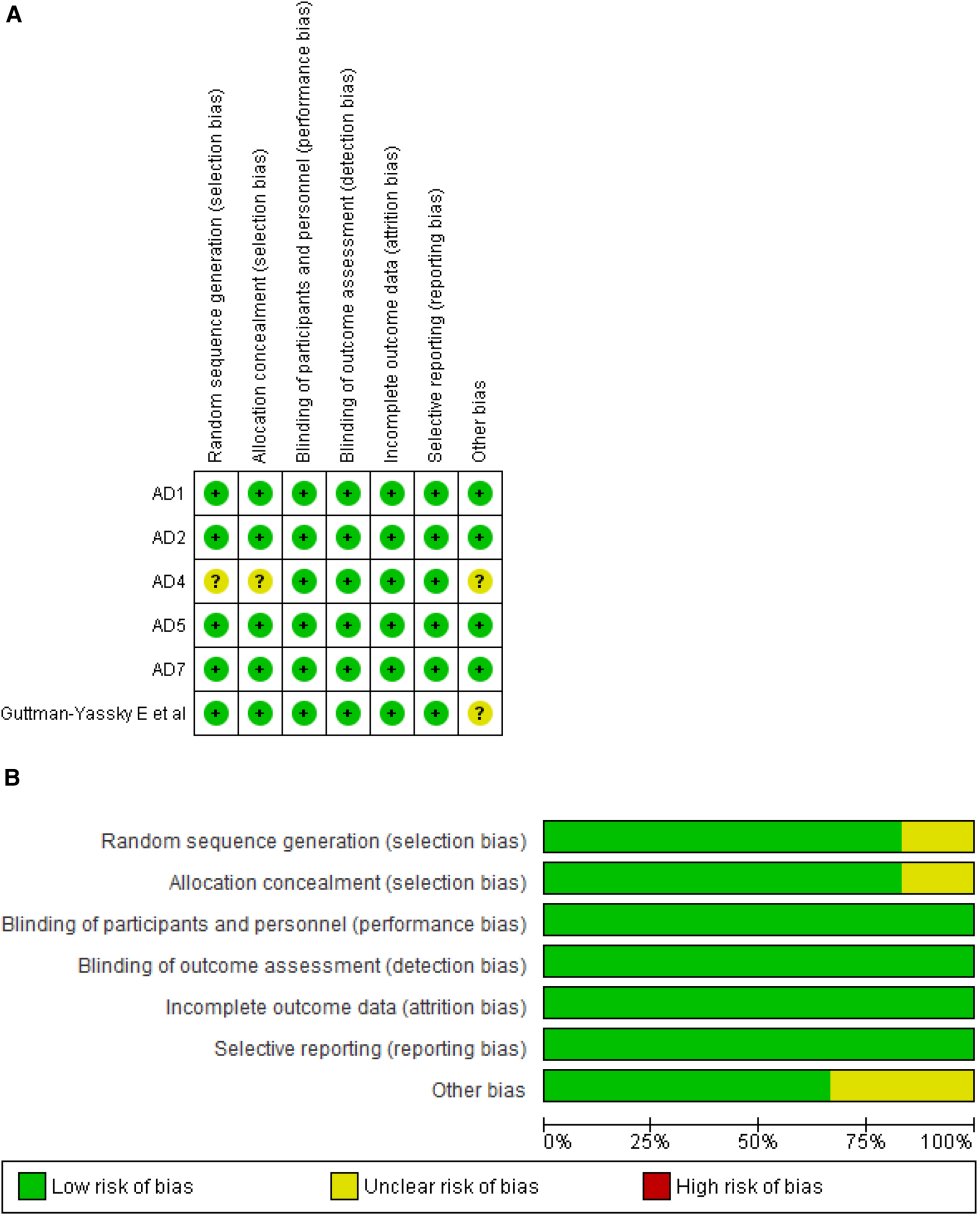
**(A)** Risk of bias summary. **(B)** Risk of bias graph.

We assessed the methodological integrity of six RCTs employing the Cochrane Risk of Bias Tool. All trials demonstrated superior quality, consistently showing a low risk of bias across the seven evaluated domains, which enhances the credibility of our findings. Figures 2A,B visually depict the rigorous assessment, while Supplementary Table 1 offers an in-depth account of the authorial evaluations for each study, furnishing a detailed perspective on the evaluation process.

### Primary outcome

#### IGA score of 0 or 1

**Figure 3:**
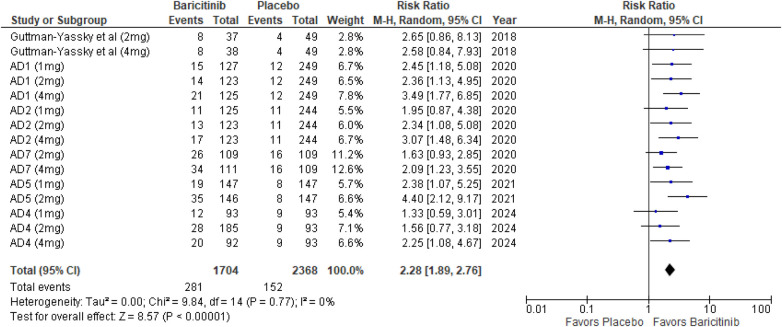
Forest plot for IGA score of 0 or 1.

The meta-analysis of studies evaluating the achievement of an IGA score of 0 or 1 demonstrated a significantly higher likelihood of success in the baricitinib group compared to the placebo group (RR: 2.28; 95% CI: 1.89–2.76; *p* < 0.00001, *I*² = 0%) (Figure 3).

In the subgroup analysis by dosage, the 1 mg dosage showed significant improvement in IGA scores (*p* = 0.0005, *I*² = 0%). The 2 mg dosage also showed a highly significant effect (*p* < 0.00001, *I*² = 12%), with low heterogeneity. Similarly, the 4 mg dosage was highly significant (*p* < 0.00001, *I*² = 0%) (Supplementary Figure 1).

Subgroup analysis based on the use of TCS vs. monotherapy showed significant outcomes in both groups. The addition of TCS significantly improved IGA scores (*p* < 0.00001, *I*² = 0%). Monotherapy without TCS was also highly effective (*p* < 0.00001, *I*² = 0%) (Supplementary Figure 2).

### Secondary outcomes

#### EASI

##### EASI 50

**Figure 4:**
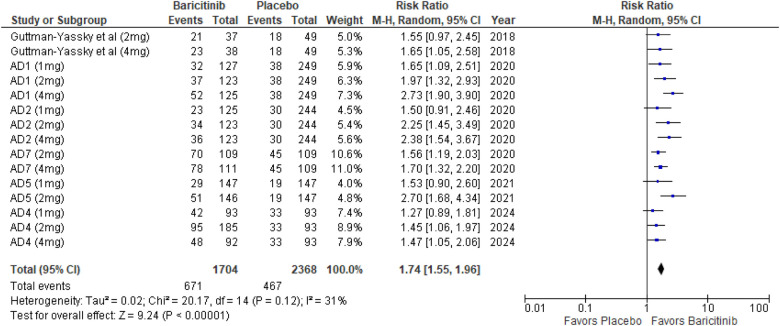
Forest plot for EASI 50.

The meta-analysis for EASI 50 demonstrated a significant benefit in the baricitinib group compared to the placebo group (RR: 1.74; 95% CI: 1.55–1.96; *p* < 0.00001, *I*² = 31%) (Figure 4).

In the subgroup analysis by dosage, the 1 mg dosage showed significant improvement (*p* = 0.0008, *I*² = 0%), indicating low heterogeneity. The 2 mg dosage also showed a highly significant effect (*p* < 0.00001, *I*² = 33%), with low heterogeneity. The 4 mg dosage showed a highly significant effect as well (*p* < 0.00001, *I*² = 52%), indicating moderate heterogeneity (Supplementary Figure 3).

Subgroup analysis based on the use of TCS vs. monotherapy showed significant outcomes in both groups. The addition of TCS significantly improved EASI 50 scores (*p* < 0.00001, *I*² = 0%), indicating low heterogeneity. Monotherapy without TCS was also highly effective (*p* < 0.00001, *I*² = 13%), with low heterogeneity (Supplementary Figure 4).

##### EASI 75

**Figure 5:**
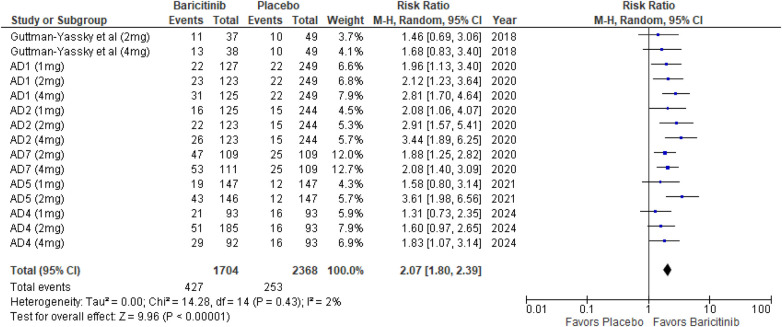
Forest plot for EASI 75.

The meta-analysis for EASI 75 revealed a significant advantage in the baricitinib group compared to the placebo group (RR: 2.07; 95% CI: 1.80–2.39; *p* < 0.00001, *I*² = 2%) (Figure 5).

In the subgroup analysis by dosage, the 1 mg dosage showed a statistically significant effect (*p* = 0.0007, *I*² = 0%), reflecting low heterogeneity. The 2 mg dosage also demonstrated a highly significant effect (*p* < 0.00001, *I*² = 24%), with low heterogeneity. The 4 mg dosage showed a highly significant result as well (*p* < 0.00001, *I*² = 1%), indicating minimal heterogeneity (Supplementary Figure 5).

Subgroup analysis comparing treatments with and without TCS showed significant effects in both scenarios. The use of TCS resulted in highly significant outcomes (*p* < 0.00001, *I*² = 0%), with no heterogeneity. Monotherapy also achieved highly significant results (*p* < 0.00001, *I*² = 0%), indicating uniformity across studies (Supplementary Figure 6).

##### EASI 90

**Figure 6:**
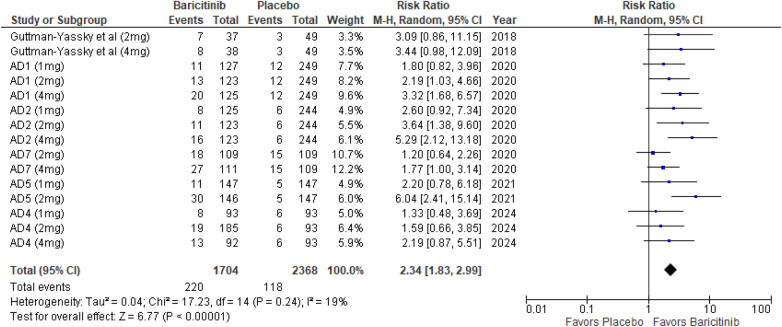
Forest plot for EASI 90.

The meta-analysis for EASI 90 indicated a significant benefit of Baricitinib compared to placebo (RR: 2.34; 95% CI: 1.83–2.99; *p* < 0.00001, *I*² = 19%) (Figure 6).

In the subgroup analysis by dosage, the 1 mg dosage showed a significant effect (*p* = 0.008, *I*² = 0%), with low heterogeneity. The 2 mg dosage demonstrated a significant effect with moderate heterogeneity (*p* = 0.0008, *I*² = 51%). The 4 mg dosage also showed a significant effect (*p* < 0.00001, *I*² = 18%), with low heterogeneity (Supplementary Figure 7).

In the subgroup analysis for Baricitinib with and without TCS, both approaches were effective. The addition of TCS yielded significant results (*p* = 0.0008, *I*² = 0%), with no heterogeneity. Monotherapy with Baricitinib also resulted in a highly significant effect (*p* < 0.00001, *I*² = 0%), indicating consistent results across studies (Supplementary Figure 8).

##### DLQI

**Figure 7:**
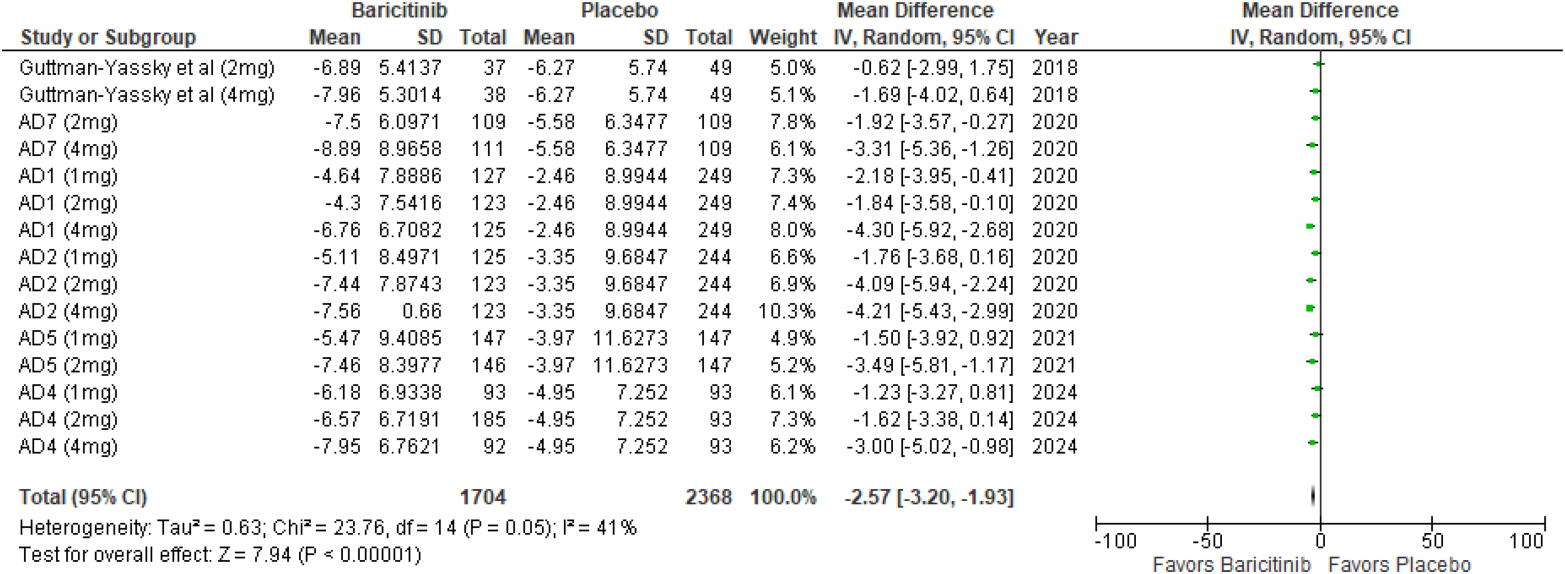
Forest plot for change from baseline in DLQI score.

The meta-analysis for DLQI showed a significant improvement with Baricitinib compared to placebo (MD: −2.57; 95% CI: −3.20 to −1.93; *p* < 0.00001, *I*² = 41%) (Figure 7).

In the subgroup analysis by dosage, the 1 mg dosage resulted in a significant effect (*p* = 0.0007, *I*² = 0%), reflecting low heterogeneity. The 2 mg dosage also showed a significant effect (*p* < 0.00001, *I*² = 34%), with low to moderate heterogeneity. The 4 mg dosage demonstrated a significant effect (*p* < 0.00001, *I*² = 14%), indicating low heterogeneity (Supplementary Figure 9).

In the subgroup analysis for Baricitinib with and without TCS, both showed significant improvements. The addition of TCS had a highly significant effect (*p* < 0.00001, *I*² = 0%), with no heterogeneity. Monotherapy without TCS was also highly significant (*p* < 0.00001, *I*² = 46%), with moderate heterogeneity (Supplementary Figure 10).

##### SCORAD

##### SCORAD 75

**Figure 8:**
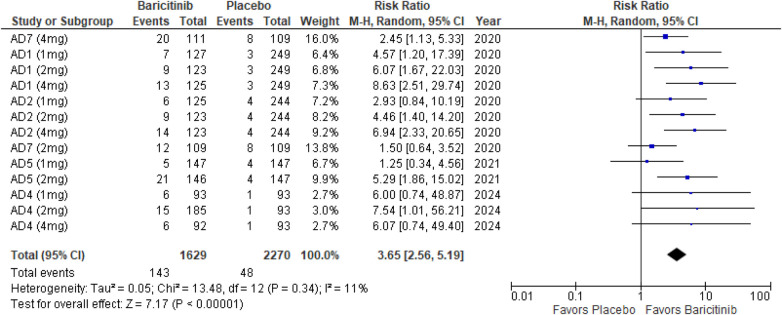
Forest plot for SCORAD 75.

The meta-analysis for SCORAD 75 showed a significant improvement with Baricitinib compared to placebo (RR: 3.65; 95% CI: 2.56–5.19; *p* < 0.00001, *I*² = 11%) (Figure 8).

In the subgroup analysis by dosage, the 1 mg dosage demonstrated a significant effect (*p* = 0.004, *I*² = 0%), with low heterogeneity. The 2 mg dosage also showed a significant result (*p* < 0.0001, *I*² = 32%), indicating low heterogeneity. The 4 mg dosage yielded a highly significant effect (*p* < 0.00001, *I*² = 27%), with low heterogeneity (Supplementary Figure 1^1^).

In the subgroup analysis for Baricitinib with and without TCS, both showed favorable outcomes. The addition of TCS had a significant effect (*p* = 0.0008, *I*² = 2%), with low heterogeneity. Monotherapy with Baricitinib also resulted in a highly significant effect (*p* < 0.00001, *I*² = 0%), with no heterogeneity (Supplementary Figure 1^2^).

##### SCORAD 90

**Figure 9:**
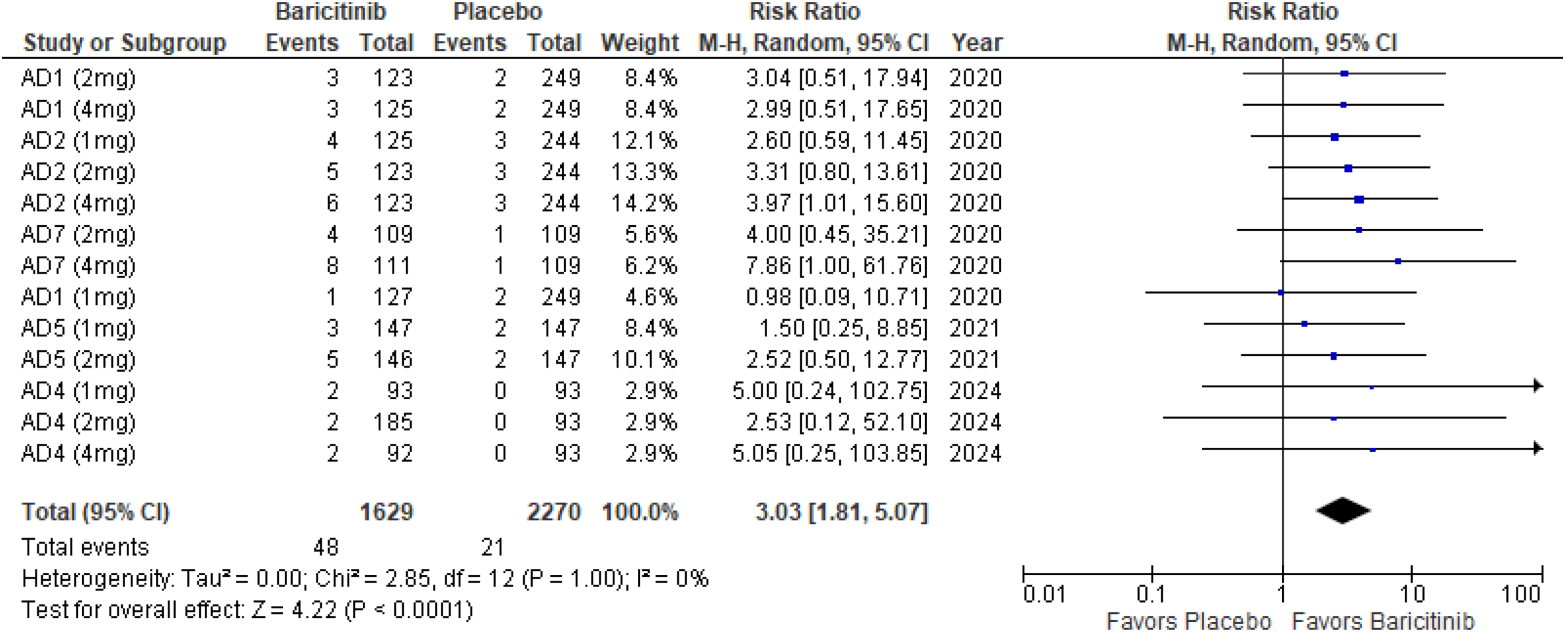
Forest plot for SCORAD 90.

The meta-analysis for SCORAD 90 demonstrated a significant improvement with Baricitinib compared to placebo (RR: 3.03; 95% CI: 1.81–5.07; *p* < 0.0001, *I*² = 0%) (Figure 9).

In the subgroup analysis by dosage, the 1 mg dosage did not show a significant effect (*p* = 0.16, *I*² = 0%), while both the 2 mg (*p* = 0.007, *I*² = 0%) and 4 mg (*p* = 0.002, *I*² = 0%) dosages demonstrated significant effects, with no heterogeneity across these analyses (Supplementary Figure 1^3^).

Subgroup analysis of Baricitinib with and without TCS indicated that both approaches were effective. The addition of TCS showed a significant result (*p* = 0.006, *I*² = 0%), with no heterogeneity. Monotherapy with Baricitinib also demonstrated a significant effect (*p* = 0.0009, *I*² = 0%), with no heterogeneity observed (Supplementary Figure 1^4^).

##### Itch NRS

**Figure 10:**
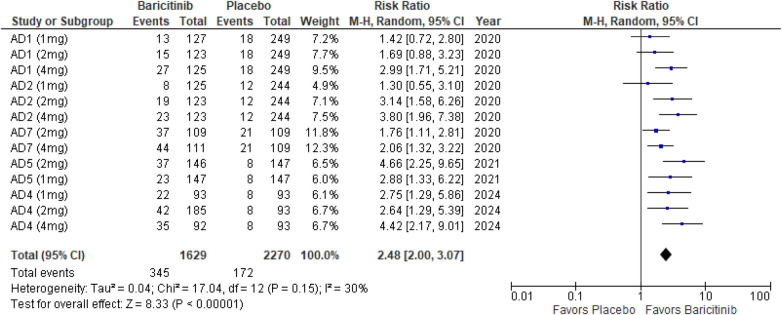
Forest plot for itch NRS.

The meta-analysis for itch NRS indicated a significant improvement with Baricitinib compared to placebo (RR: 2.48; 95% CI: 2.00–3.07; *p* < 0.00001, *I*² = 30%) (Figure 10).

In the subgroup analysis by dosage, the 1 mg dosage demonstrated a significant effect (*p* = 0.001, *I*² = 13%), with low heterogeneity. The 2 mg dosage also showed a highly significant effect (*p* < 0.00001, *I*² = 41%), with low to moderate heterogeneity. The 4 mg dosage yielded a significant result (*p* < 0.00001, *I*² = 30%), indicating low heterogeneity (Supplementary Figure 1^5^).

Subgroup analysis of Baricitinib with and without TCS revealed significant results in both groups. The use of TCS resulted in a significant improvement (*p* < 0.00001, *I*² = 23%), with low heterogeneity. Monotherapy with Baricitinib also showed significant outcomes (*p* < 0.00001, *I*² = 39%), with low to moderate heterogeneity (Supplementary Figure 1^6^).

#### Skin infections requiring antibiotic treatment

**Figure 11:**
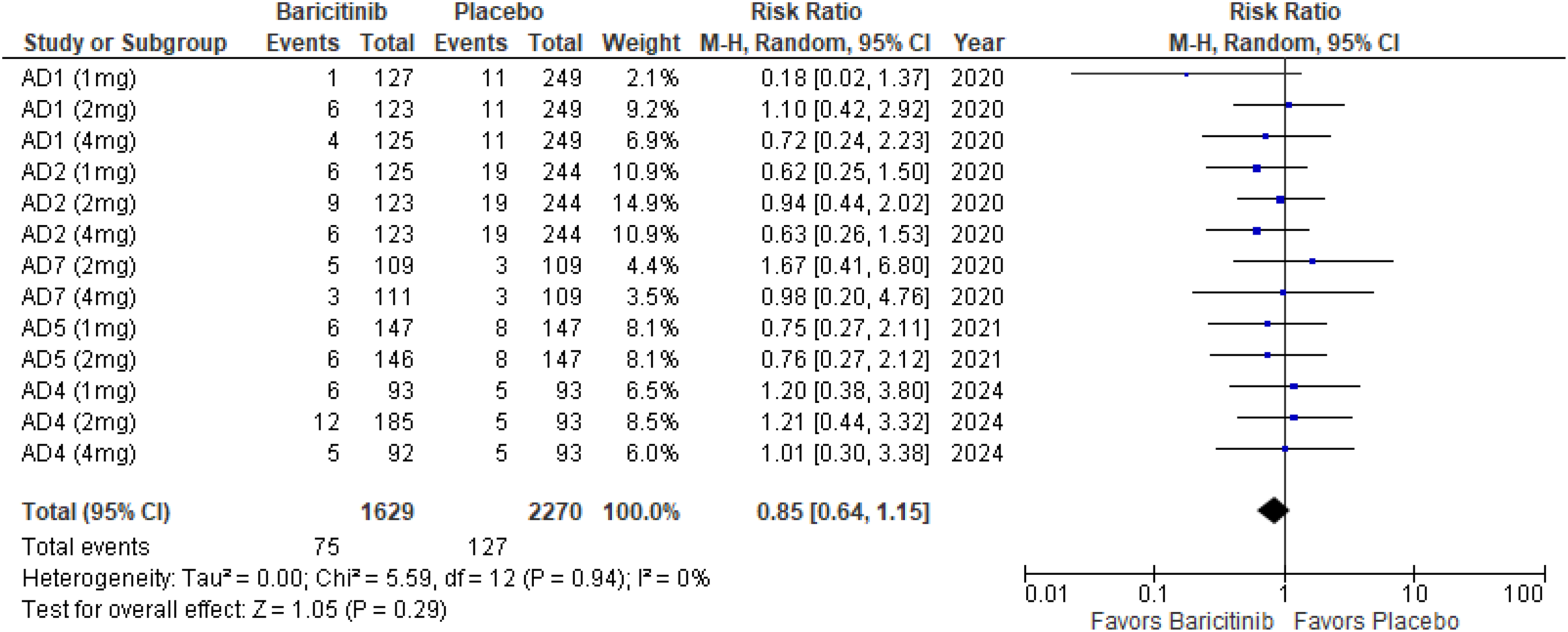
Forest plot for skin infections requiring antibiotic treatment.

The meta-analysis for skin infections requiring antibiotic treatment showed no significant difference between Baricitinib and placebo (RR: 0.85; 95% CI: 0.64–1.15; *p* = 0.29, *I*² = 0%) (Figure 11).

In the subgroup analysis by dosage, none of the dosages showed a significant effect. The 1 mg dosage had a *p*-value of 0.20 (*I*² = 0%), the 2 mg dosage had a *p*-value of 0.88 (*I*² = 0%), and the 4 mg dosage had a *p*-value of 0.35 (*I*² = 0%), all indicating no heterogeneity (Supplementary Figure 1^7^).

Subgroup analysis for Baricitinib with and without TCS also showed no significant differences. With TCS, the *p*-value was 0.53 (*I*² = 0%), and for monotherapy, the *p*-value was 0.10 (*I*² = 0%), both reflecting no heterogeneity across studies (Supplementary Figure 1^8^).

#### Skin pain NRS

**Figure 12:**
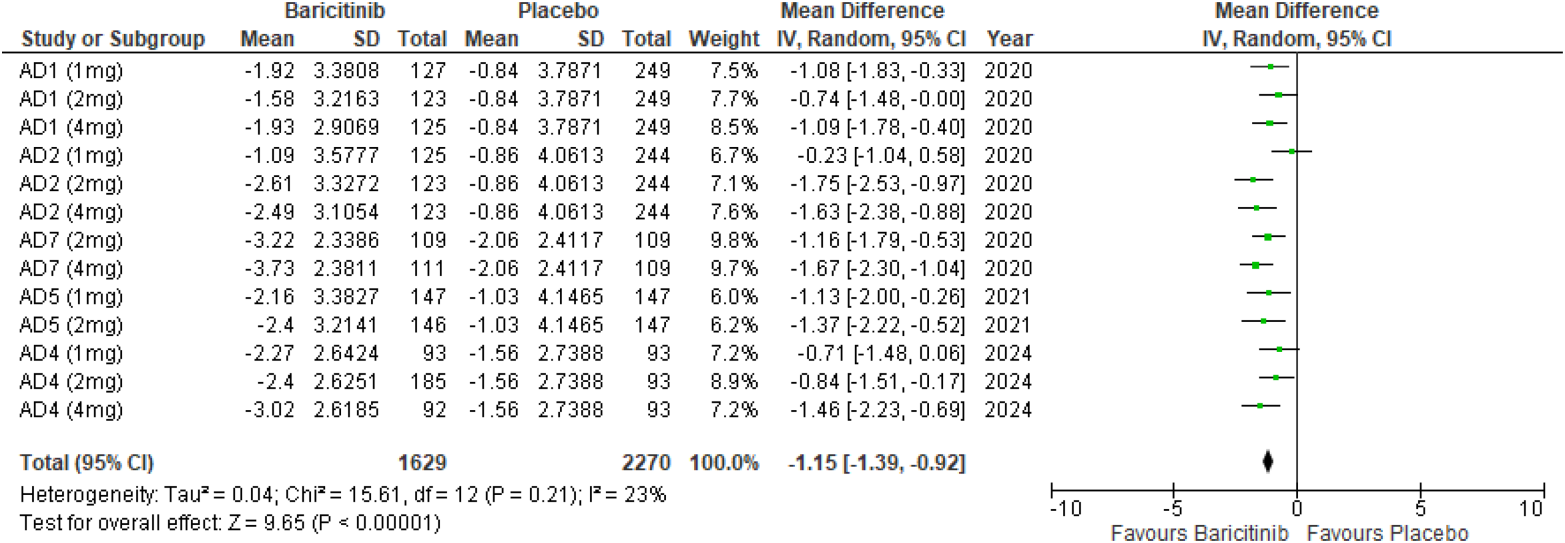
Forest plot for skin Pain NRS.

The meta-analysis for skin pain NRS demonstrated a significant reduction in pain with Baricitinib compared to placebo (MD: −1.15; 95% CI: −1.39 to −0.92; *p* < 0.00001, *I*² = 23%) (Figure 12).

In the subgroup analysis by dosage, the 1 mg dosage showed a significant reduction in skin pain (*p* = 0.0001, *I*² = 2%), with low heterogeneity. The 2 mg dosage also showed a significant effect (*p* < 0.00001, *I*² = 12%), with low heterogeneity. The 4 mg dosage yielded a significant result as well (*p* < 0.00001, *I*² = 0%), with no heterogeneity (Supplementary Figure 1^9^).

Subgroup analysis of Baricitinib with and without TCS showed that both approaches significantly reduced skin pain. The use of TCS resulted in a significant reduction (*p* < 0.00001, *I*² = 23%), with low heterogeneity. Monotherapy with Baricitinib also showed a significant reduction in skin pain (*p* < 0.00001, *I*² = 32%), with low to moderate heterogeneity (Supplementary Figure 20).

#### BSA affected

**Figure 13:**
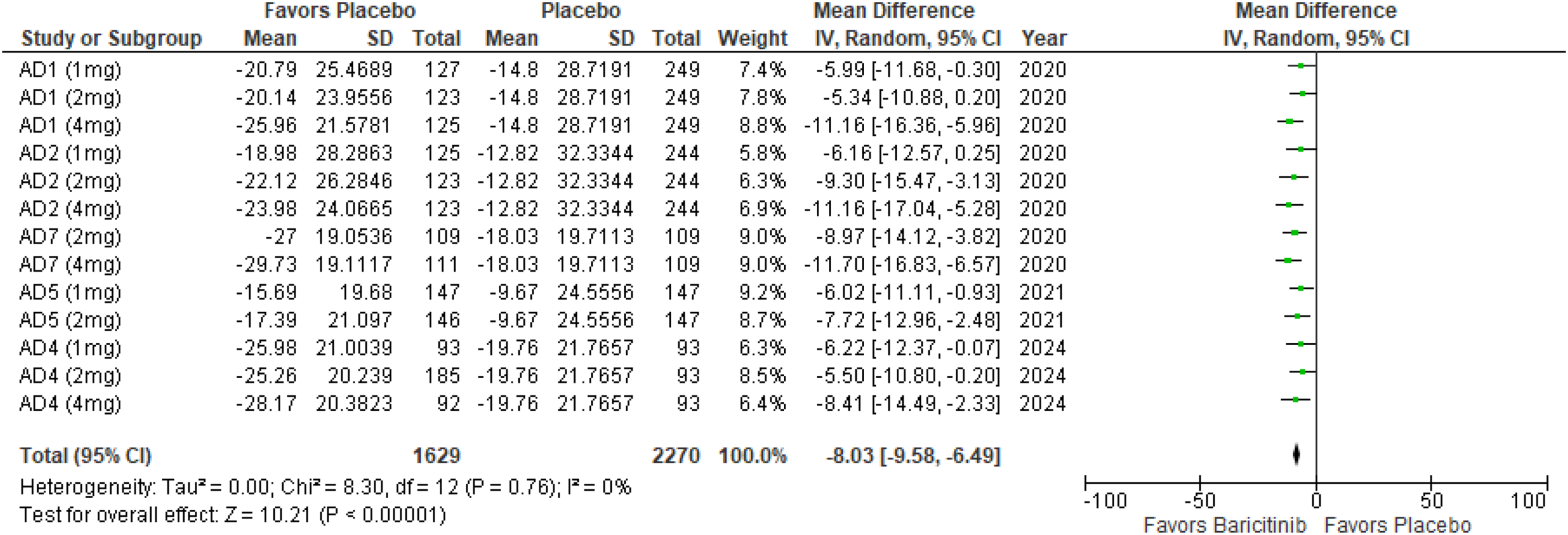
Forest plot for BSA affected.

The meta-analysis for BSA affected showed a significant reduction with Baricitinib compared to placebo (MD: −8.03; 95% CI: −9.58 to −6.49; *p* < 0.00001, *I*² = 0%) (Figure 13).

In the subgroup analysis by dosage, the 1 mg dosage demonstrated a significant reduction in BSA affected (*p* < 0.0001, *I*² = 0%), with no heterogeneity. The 2 mg dosage also showed a significant effect (*p* < 0.00001, *I*² = 0%), with no heterogeneity. The 4 mg dosage similarly demonstrated a significant reduction (*p* < 0.00001, *I*² = 0%), with no heterogeneity (Supplementary Figure 2^1^).

Subgroup analysis for Baricitinib with and without TCS indicated significant improvements in both cases. The use of TCS resulted in a significant reduction in BSA affected (*p* < 0.00001, *I*² = 0%), with no heterogeneity. Monotherapy with Baricitinib also showed a significant reduction (*p* < 0.00001, *I*² = 0%), with no heterogeneity across studies (**S**upplementary Figure 2^2^).

##### POEM

**Figure 14:**
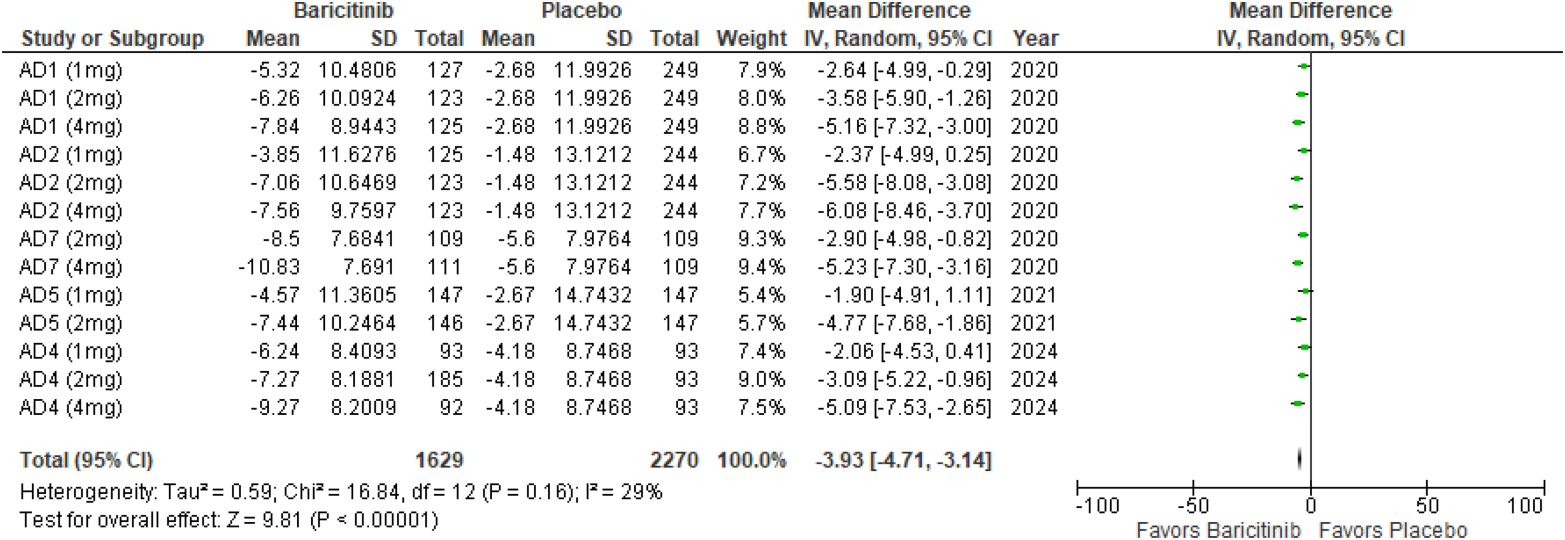
Forest plot for POEM.

The meta-analysis for POEM demonstrated a significant reduction in scores with Baricitinib compared to placebo (MD: −3.93; 95% CI: −4.71 to −3.14; *p* < 0.00001, *I*² = 29%) (Figure 14).

In the subgroup analysis by dosage, the 1 mg dosage showed a significant reduction in POEM scores (*p* = 0.0005, *I*² = 0%), with no heterogeneity. The 2 mg dosage also demonstrated a significant effect (*p* < 0.00001, *I*² = 0%), with no heterogeneity. Similarly, the 4 mg dosage resulted in a significant reduction (*p* < 0.00001, *I*² = 0%), with no heterogeneity (Supplementary Figure 2^3^).

Subgroup analysis of Baricitinib with and without TCS indicated significant improvements in both groups. The use of TCS resulted in a significant reduction (*p* < 0.00001, *I*² = 32%), with low heterogeneity. Monotherapy with Baricitinib also showed a significant reduction in POEM scores (*p* < 0.00001, *I*² = 33%), with low heterogeneity (Supplementary Figure 2^4^).

##### TEAEs

**Figure 15:**
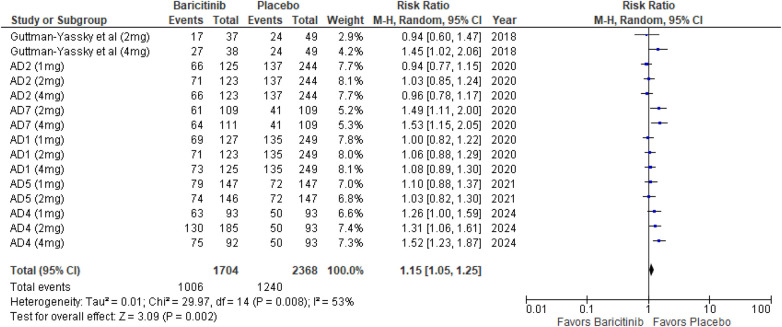
Forest plot for TEAEs.

The analysis for TEAEs showed a significant increase with Baricitinib compared to placebo, with a *p*-value of 0.002 (RR: 1.15; 95% CI: 1.05–1.25, *I*² = 53%), indicating moderate heterogeneity (Figure 15).

In the subgroup analysis by dosage, the 1 mg dosage did not show a significant effect (*p* = 0.38, *I*² = 22%), with low heterogeneity. The 2 mg dosage reached significance (*p* = 0.04, *I*² = 36%), with low heterogeneity. The 4 mg dosage also reached significance (*p* = 0.03, *I*² = 73%), indicating substantial heterogeneity (Supplementary Figure 2^5^).

Subgroup analysis for Baricitinib with and without TCS revealed that TEAEs were significantly higher with TCS (*p* < 0.00001, *I*² = 0%), with no heterogeneity. Monotherapy showed no significant difference (*p* = 0.53, *I*² = 0%), with no heterogeneity **(**Supplementary Figure 2^6^).

## Discussion

The findings of this meta-analysis provide compelling evidence that baricitinib, across various dosages, is significantly more effective than placebo in improving multiple clinical outcomes for patients with AD. The intervention consistently demonstrated superior results in achieving key endpoints such as IGA scores, EASI, SCORAD, DLQI, and POEM, with low to moderate heterogeneity, suggesting the robustness of these effects. Subgroup analyses further confirmed the efficacy of baricitinib both as monotherapy and in combination with topical corticosteroids. Notably, while the incidence of skin infections requiring antibiotic treatment did not significantly differ between the baricitinib and placebo groups, there was a trend towards increased treatment-emergent adverse events associated with baricitinib, particularly when combined with topical corticosteroids. These results underscore the potential of baricitinib as a therapeutic option for AD, while also highlighting the need for careful consideration of adverse effects, especially in combination therapies.

The pathogenesis of AD is multifaceted, involving various environmental triggers and genetic susceptibility factors. This complex nature of the disease combines with immune dysregulation, where Th2 cytokines like IL-4, IL-13, and IL-31 drive inflammation, IgE production, and itching. Additional cytokines, including IL-22 from Th22 cells, contribute to skin barrier dysfunction, while IL-17 and IL-23 from Th17 cells exacerbate chronic inflammation. Elevated IL-1, IL-6, TNF-alpha, and dysregulated IL-10 further amplify the immune response. Moreover, IL-33 and TSLP play key roles in initiating inflammation in response to environmental factors, highlighting the intricate cytokine network that underpins the chronic and relapsing nature of the disease (21). Many of these processes depend on the JAK–STAT pathway for signal transduction, which is essential for mediating the effects of key cytokines that bind to immune cells, keratinocytes, and peripheral sensory neurons, thereby propagating inflammation and itch. This pathway also plays a role in augmenting the Th2 cell response, suppressing regulatory T cells, and activating eosinophils, all of which are critical in the pathogenesis of AD (8).

Baricitinib is a first-generation, small-molecule, reversible inhibitor of the JAK family, specifically targeting JAK1 and JAK2 subtypes (22). The exact mechanism involves baricitinib competitively inhibiting the ATP-binding site of JAK enzymes. By blocking this site, baricitinib prevents the phosphorylation of tyrosine residues on cytokine receptors, which is a crucial step in the activation of the JAK/STAT signaling pathway. Normally, when cytokines bind to their respective receptors, JAK enzymes are activated and phosphorylate specific receptor tyrosine residues. These phosphorylated residues then serve as docking sites for STAT proteins, which get phosphorylated, dimerize, and translocate to the nucleus to regulate gene expression (23). This interruption in cytokine signaling effectively reduces the inflammatory response mediated by cytokines such as IL-4, IL-13, IL-31, IL-22, and IFN-*γ*, which are critical in conditions like AD, psoriasis, and alopecia areata (AA) (24). Additionally, baricitinib is approved for the treatment of rheumatoid arthritis (25).

We chose IGA, EASI, DLQI and SCORAD as efficacy metrics due to their prominence as the most frequently employed disease severity assessment tools in clinical research (26). The IGA evaluates overall AD severity on a 5-point scale from 0 (clear skin) to 4 (severe disease), based on erythema, papulation/induration, oozing/crusting, and lichenification (27). The EASI measures disease extent and severity of four clinical signs—erythema, edema/papulation, excoriation, and lichenification—each rated from 0 (none) to 3 (severe) across four body regions: head/neck, trunk, upper limbs, and lower limbs (28). The SCORAD index uses the rule of nines to assess disease extent and rates six clinical features—erythema, edema/papulation, oozing/crusting, excoriation, lichenification, and dryness—on a scale from 0 (absent) to 3 (severe) (29). The DLQI is a self-administered, 10-question survey that evaluates quality of life across six domains: symptoms, daily activities, leisure, work/school, relationships, and treatment, and is another patient-reported outcome assessing various aspects of a patient's health-related quality of life (30).

Our meta-analysis revealed that patients receiving Baricitinib had significantly higher rates of achieving key outcomes, including IGA scores of 0 or 1, EASI 50, EASI 75, EASI 90, DLQI improvements, SCORAD 75, and SCORAD 90 compared to placebo. Subgroup analyses further demonstrated that all three Baricitinib doses (1, 2, and 4 mg) were effective across these outcomes, with higher doses generally showing stronger effects. Additionally, analyses of patients using Baricitinib with or without TCS indicated significant improvements in both groups, with consistent results observed across various dosages and outcomes, showing minimal to moderate heterogeneity across studies.

The Itch NRS and Skin Pain NRS are both participant-administered 11-point scales, where 0 indicates “no itch/pain” and 10 indicates “worst imaginable itch/pain” (31). The POEM is a 7-item self-assessment questionnaire that measures symptoms like dryness, itching, and sleep loss, with scores ranging from 0 (no days) to 4 (every day). The total POEM score, summing these items, ranges from 0 (no disease) to 28 (severe disease) (32). Additionally, the BSA affected by AD is assessed across four body regions (33). The meta-analysis demonstrated that Baricitinib led to significant improvements across multiple measures, including itch severity, skin pain, BSA affected, and POEM scores, when compared to placebo. Subgroup analysis by dosage revealed that all three dosages (1 mg, 2 mg, and 4 mg) were effective, with varying degrees of heterogeneity across the outcomes. Additionally, the analysis of Baricitinib with and without TCS showed significant benefits in both scenarios, indicating that the treatment effectively reduced symptoms regardless of adjunctive therapy.

Recent safety concerns have emerged regarding JAK inhibitors, particularly in relation to serious cardiovascular events and malignancies. These concerns were highlighted by a large, randomized safety trial involving baricitinib in patients with rheumatoid arthritis. In a long-term study of 3,770 rheumatoid arthritis patients treated with baricitinib, the standardized incidence ratios for severe infections, herpes zoster, and major adverse cardiovascular events (MACEs) were 2.6, 3.0, and 0.5, respectively. The incidence of malignant tumors was 0.6 in the first 48 weeks and remained stable in subsequent observations (34). Baricitinib is also linked to a higher incidence of adverse events, particularly herpes zoster, due to its inhibition profile targeting JAK1 and JAK2. These JAK enzymes play a critical role in immune system regulation, especially in controlling viral infections like varicella-zoster virus, the reactivation of which leads to herpes zoster. Baricitinib's potent inhibition of JAK2, compared to other JAK inhibitors, disrupts Type I and Type II interferon signaling, both of which are essential for the immune system to prevent VZV reactivation. Additionally, the risk of adverse events may increase due to dose-dependent “pan-JAK” inhibition, which can affect other JAK pathways like TYK2. This broader inhibition compromises the body's immune defenses. Furthermore, baricitinib reduces natural killer (NK) cell counts, cells that are crucial for controlling viral infections. The combined effects of interferon suppression, NK cell reduction, and broader JAK inhibition explain the association of baricitinib with a higher risk of infections, particularly herpes zoster (35). Regarding safety outcomes, we assessed the proportions of patients who experienced TEAEs and those who developed skin infections necessitating antibiotic therapy. The analysis of TEAEs showed a significant increase with Baricitinib compared to placebo. In dosage-based subgroup analysis, the 1 mg dosage did not show a significant effect, while the 2 mg and 4 mg dosages both reached statistical significance, with varying levels of heterogeneity across groups. The addition of TCS significantly elevated the incidence of TEAEs, while monotherapy did not reveal any significant differences. For skin infections requiring antibiotic treatment, the meta-analysis indicated no significant difference between Baricitinib and placebo across all dosages and in subgroup analyses with or without TCS, with no heterogeneity observed. In this analysis, three studies evaluated baricitinib in combination with TCS, while the remaining three studies focused on baricitinib monotherapy. The results indicated that baricitinib monotherapy was equally effective as the combination therapy in improving patient outcomes, suggesting that the addition of TCS may not provide substantial added benefit. Notably, baricitinib monotherapy was associated with fewer treatment-emergent adverse events compared to the combination therapy, highlighting its potential as a safer alternative. These findings underscore the viability of monotherapy for patients who may be at risk for adverse effects from additional treatments.

This meta-analysis represents the first in-depth evaluation of six recent RCTs assessing the efficacy and safety of Baricitinib in treating AD, uniquely comparing both Baricitinib monotherapy and its combination with TCS against placebo. The inclusion of rigorous subgroup analyses strengthens the reliability of these findings, offering nuanced insights into drug performance. Additionally, the low heterogeneity observed across outcomes further supports the consistency and validity of the results. Despite the valuable insights provided by this meta-analysis, several limitations must be acknowledged. First, despite the inclusion of six randomized controlled trials, the overall number of studies remains limited, potentially impacting the generalizability of the findings. Additionally, while all included studies had a uniform follow-up duration of 16 weeks, this relatively short period restricts the evaluation of long-term efficacy and safety of baricitinib for AD. Concerns regarding long-term safety, such as serious infections and hematologic abnormalities, are not fully addressed due to this limited follow-up. The sample sizes within the included trials were also relatively small, which may influence the robustness of the results. Furthermore, the absence of an active comparator group limits the ability to benchmark baricitinib's performance against other current treatment options. One of the phase 3 randomized controlled trials included in the analysis has not yet been published, with results only available on ClinicalTrials.gov, which could affect the comprehensiveness of the data. Additionally, the included studies assessed various doses of baricitinib, either as monotherapy or in combination with topical corticosteroids. However, during subgroup analyses, studies were often mixed, complicating the ability to draw firm conclusions about specific combinations. Future research should include larger scale randomized controlled trials with extended follow-up periods, active comparator groups, and a focus on long-term safety outcomes to further validate these findings and assess long-term effects.

## Conclusion

In summary, this meta-analysis highlights the efficacy of baricitinib in treating moderate-to-severe AD. Both monotherapy and combination with topical corticosteroids significantly improved clinical outcomes, including IGA scores and various EASI metrics, with consistent effects across different dosages. Although baricitinib showed substantial efficacy, a significant increase in TEAEs was observed, particularly when used with corticosteroids. Dosages of 2 mg and 4 mg also showed statistical significance. These findings support baricitinib as a viable treatment option, warranting careful consideration of its safety profile in clinical decision-making.

## Data Availability

The original contributions presented in the study are included in the article/Supplementary Material, further inquiries can be directed to the corresponding author.
