# Supplementary material for: Baricitinib as monotherapy and with topical corticosteroids in moderate-to-severe atopic dermatitis: a systematic review and meta-analysis of dose-response
Source: Front Allergy. 2024 Nov 14;5:1486271. doi: 10.3389/falgy.2024.1486271 (PMC11602504; doi:10.3389/falgy.2024.1486271)
Supplement: Supplementary file 1 [file Datasheet1.docx]

**Supplementary Appendix**

**Supplementary Table 1.** Risk of Bias Assessment Table

|  | Cochrane Risk-of-Bias Tool | | |
| --- | --- | --- | --- |
|  | Bias | Risk of bias | Author judgement |
| Simpson EL et al (AD5) 2021 NCT03435081 | Random sequence generation (selection bias) | Low Risk | The study involved randomization (1:1:1) to placebo, baricitinib 1 mg, or baricitinib 2 mg. Although the exact method of randomization was not detailed, the use of random assignment typically indicates low risk of selection bias if implemented properly. |
|  | Allocation concealment (selection bias) | Low Risk | The study is double-blinded, which suggests that allocation concealment was maintained. Both participants and researchers were likely unaware of treatment assignments, reducing the risk of selection bias. |
|  | Blinding of participants and personnel (performance bias) | Low Risk | The study was double-blinded, ensuring that both participants and investigators did not know the treatment assignments. This helps minimize performance bias by preventing differential treatment based on knowledge of the intervention. |
|  | Blinding of outcome assessment (detection bias) | Low Risk | The study design indicates that outcome assessors were blinded, which helps in minimizing detection bias as the assessors’ knowledge of the treatment group would not affect outcome assessments. |
|  | Incomplete outcome data (attrition bias) | low Risk | The study included all randomized patients who received at least one dose of the study drug in the safety analyses and applied intention-to-treat principles for efficacy analyses. This approach reduces attrition bias by accounting for missing data and participants lost to follow-up. |
|  | Selective reporting (reporting bias) | Low Risk | . The study revised its primary endpoint before database lock, with detailed justifications provided. The transparent reporting and adjustments suggest a low risk of selective reporting bias. |
|  | Other bias | Low Risk | The study adhered to ethical guidelines and included comprehensive safety assessments. There is no indication of other biases based on the provided information. |
| Reich K et al (AD7) 2020  NCT03733301 | Random sequence generation (selection bias) | Low Risk | The study employed computer-generated random sequencing for the random allocation of patients in a 1:1:1 ratio. This method ensures that the randomization process was adequately conducted, reducing the likelihood of selection bias. |
|  | Allocation concealment (selection bias) | Low Risk | Allocation concealment was achieved through the use of double-blind investigational product tablets provided to patients at each visit. The concealment method prevented researchers and participants from knowing the treatment allocation, which minimizes the risk of selection bias. |
|  | Blinding of participants and personnel (performance bias) | Low Risk | The trial maintained a double-blind design, ensuring that both participants and personnel were unaware of the treatment assignments. This blinding helps to prevent performance bias, as expectations about treatment outcomes were minimized. |
|  | Blinding of outcome assessment (detection bias) | Low Risk | Blinded outcome assessment was conducted using standardized measures, such as the validated Investigator Global Assessment for Atopic Dermatitis (vIGA-AD). The blinding of assessors to treatment allocation reduces the potential for detection bias. |
|  | Incomplete outcome data (attrition bias) | Low Risk | The study addressed incomplete data by applying a nonresponder imputation for categorical endpoints and censored data after rescue therapy for continuous endpoints. These methods ensure that the impact of missing data is minimized, reducing the risk of attrition bias. |
|  | Selective reporting (reporting bias) | Low Risk | The primary and secondary endpoints were pre-specified and adjusted for multiplicity, as indicated by the prespecified statistical analysis plan. This approach decreases the likelihood of selective reporting bias. |
|  | Other bias | Low Risk | The study followed Good Clinical Practice guidelines, was conducted in accordance with the Declaration of Helsinki, and adhered to the CONSORT reporting guideline. These measures, along with the independent ethics review and approval process, suggest a well-conducted study with a low likelihood of other biases. |
| Guttman-Yassky E 2018  NCT02576938 | Random sequence generation (selection bias) | Low Risk | The study used blocked randomization with stratification and an interactive response technology (IRT) system, which suggests a well-controlled randomization process. |
|  | Allocation concealment (selection bias) | Low Risk | Allocation concealment was ensured by using the IRT system, and the randomization schedule was maintained by an independent entity (PAREXEL), which reduces the risk of selection bias. |
|  | Blinding of participants and personnel (performance bias) | Low Risk | The study was double-blind, with patients, investigators, and study site personnel blinded to treatment allocation, minimizing the risk of performance bias. |
|  | Blinding of outcome assessment (detection bias) | Low Risk | Outcome assessment was conducted by blinded investigators, which helps reduce detection bias. |
|  | Incomplete outcome data (attrition bias) | Low Risk | The study considered all patients who discontinued the study or treatment before week 16 as non-responders, which is a conservative approach that reduces the risk of attrition bias. |
|  | Selective reporting (reporting bias) | Low Risk | The primary and secondary outcomes were clearly defined, and statistical methods were detailed, reducing the risk of selective reporting. |
|  | Other bias | Unclear Risk | No specific details on other potential sources of bias, such as conflicts of interest or funding sources, were provided, making it difficult to fully assess other biases. |
| Simpson EL et al (AD1) NCT03334396 | Random sequence generation (selection bias) | Low Risk | Randomization in BREEZE-AD1 was stratified by geography and baseline disease severity, utilizing an interactive web response system. This indicates that a systematic and unbiased random sequence generation process was followed. |
|  | Allocation concealment (selection bias) | Low Risk | The treatment allocation process in BREEZE-AD1 was blinded, with identical placebo tablets used to maintain the blinding, ensuring that allocation concealment was adequately maintained. |
|  | Blinding of participants and personnel (performance bias) | Low Risk | Both patients and investigators in BREEZE-AD1 were blinded to the treatment allocation, reducing the potential for performance bias.. |
|  | Blinding of outcome assessment (detection bias) | Low Risk | Outcome assessments were conducted using the blinded Validated Investigator’s Global Assessment of AD (vIGA-AD) in BREEZE-AD1, minimizing detection bias. |
|  | Incomplete outcome data (attrition bias) | Low Risk | BREEZE-AD1 implemented a robust approach to handling missing data, using nonresponder imputation and MMRM analyses, which helps reduce attrition bias. |
|  | Selective reporting (reporting bias) | Low Risk | The primary and secondary endpoints in BREEZE-AD1 were clearly defined, and the statistical analysis plan was detailed, which reduces the risk of selective reporting. |
|  | Other bias | Low Risk | BREEZE-AD1 had its safety data regularly reviewed by an independent data-monitoring committee, and no other biases were evident in the design or execution of the study. |
| Simpson EL et al (AD2) NCT03334422 | Random sequence generation (selection bias) | Low Risk | Like BREEZE-AD1, BREEZE-AD2 used stratified randomization by geography and baseline disease severity with an interactive web response system, ensuring a well-controlled randomization process. |
|  | Allocation concealment (selection bias) | Low Risk | In BREEZE-AD2, the use of identical placebo tablets and blinding of treatment allocation to both patients and investigators ensured proper allocation concealment. |
|  | Blinding of participants and personnel (performance bias) | Low Risk | BREEZE-AD2 maintained blinding of both participants and study personnel, reducing the potential for performance bias like BREEZE-AD1. |
|  | Blinding of outcome assessment (detection bias) | Low Risk | As in BREEZE-AD1, outcome assessments in BREEZE-AD2 were conducted using the blinded vIGA-AD scale, reducing detection bias. |
|  | Incomplete outcome data (attrition bias) | Low Risk | BREEZE-AD2 also applied nonresponder imputation and MMRM analyses to handle missing data, which is consistent with the approach in BREEZE-AD1 and helps mitigate attrition bias. |
|  | Selective reporting (reporting bias) | Low Risk | The study design in BREEZE-AD2 included clearly defined endpoints and a comprehensive statistical analysis plan, reducing the risk of selective reporting. |
|  | Other bias | Low Risk | BREEZE-AD2 was also monitored by an independent data-monitoring committee, and no other significant biases were identified in the study design or execution. |
| NCT03428100 (AD4) | Random sequence generation (selection bias) | Unclear Risk | Only results are published on clinicaltrials.gov |
|  | Allocation concealment (selection bias) | Unclear Risk | Only results are published on clinicaltrials.gov |
|  | Blinding of participants and personnel (performance bias) | Low Risk | Only results are published on clinicaltrials.gov |
|  | Blinding of outcome assessment (detection bias) | Low Risk | Only results are published on clinicaltrials.gov |
|  | Incomplete outcome data (attrition bias) | Low Risk | Only results are published on clinicaltrials.gov |
|  | Selective reporting (reporting bias) | Low Risk | Only results are published on clinicaltrials.gov |
|  | Other bias | Unclear Risk | Only results are published on clinicaltrials.gov |

**Supplementary Figure 1: Forest plot for subgroup analysis of IGA score of 0 or 1 by dosage**

**
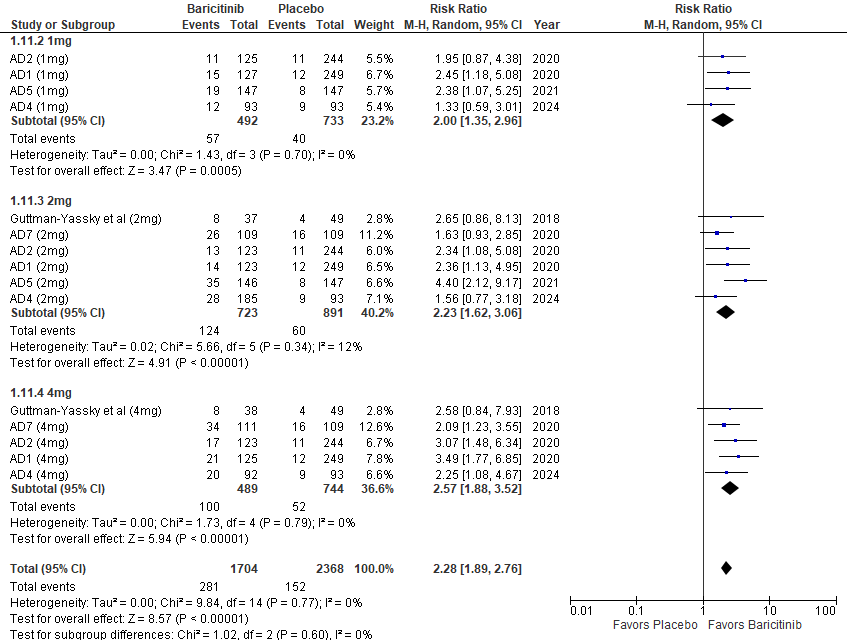
**

**Supplementary Figure 2: Forest plot for subgroup analysis of IGA score of 0 or 1 with/without TCS**


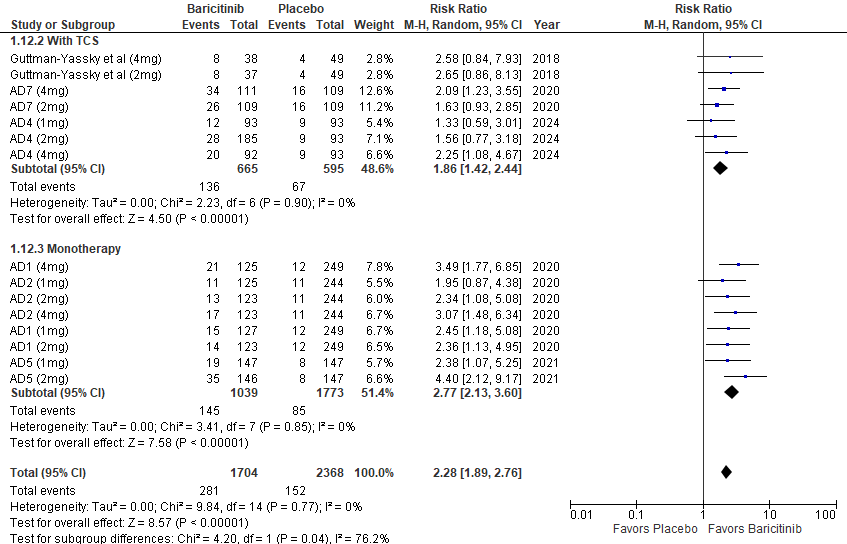


**Supplementary Figure 3: Forest plot for subgroup analysis of EASI 50 by dosage**


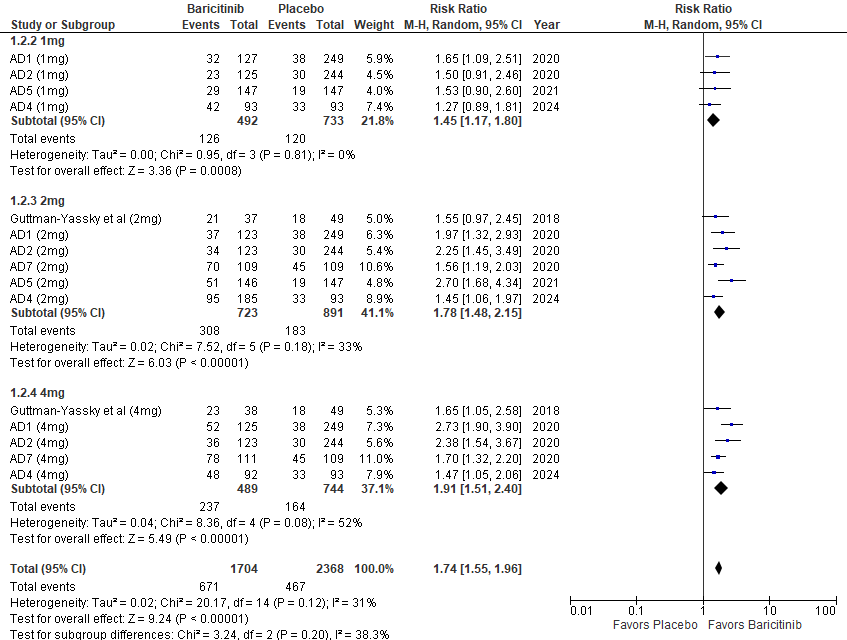


**Supplementary Figure 4: Forest plot for subgroup analysis of EASI 50 with/without TCS**

**
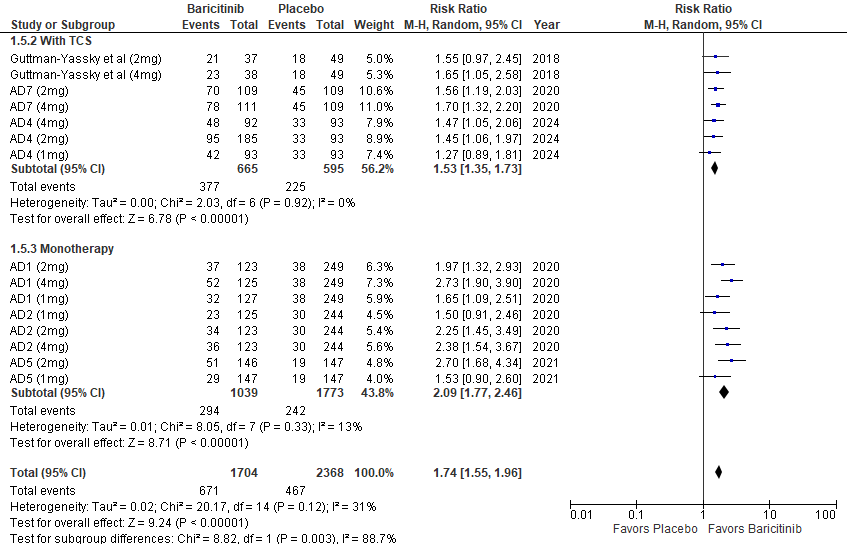
**

**Supplementary Figure 5: Forest plot for subgroup analysis of EASI 75 by dosage**


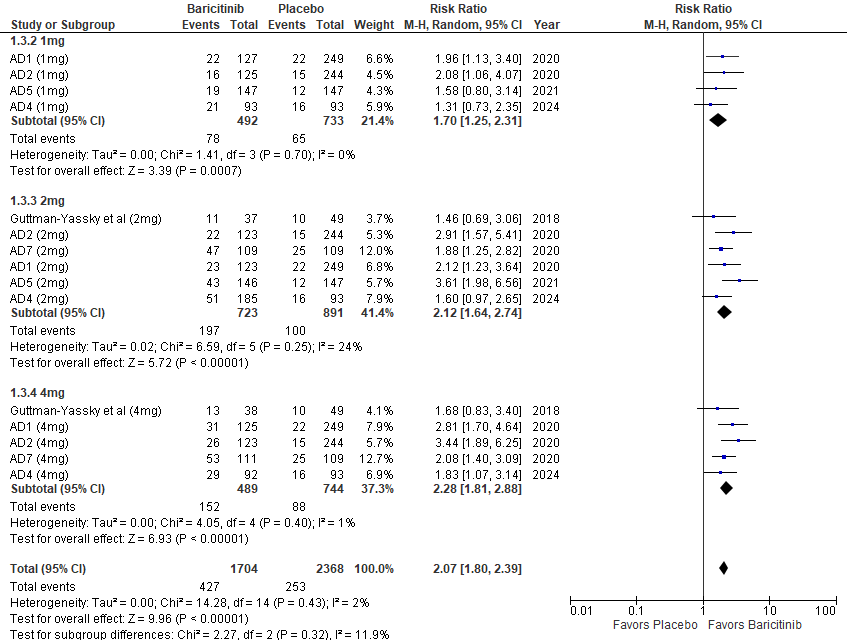


**Supplementary Figure 6: Forest plot for subgroup analysis of EASI 75 with/without TCS**


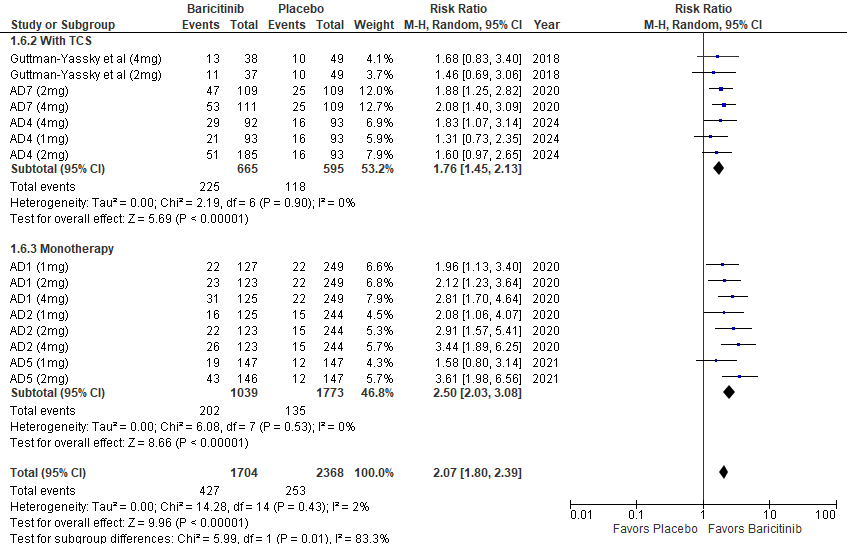


**Supplementary Figure 7: Forest plot for subgroup analysis of EASI 90 by dosage**


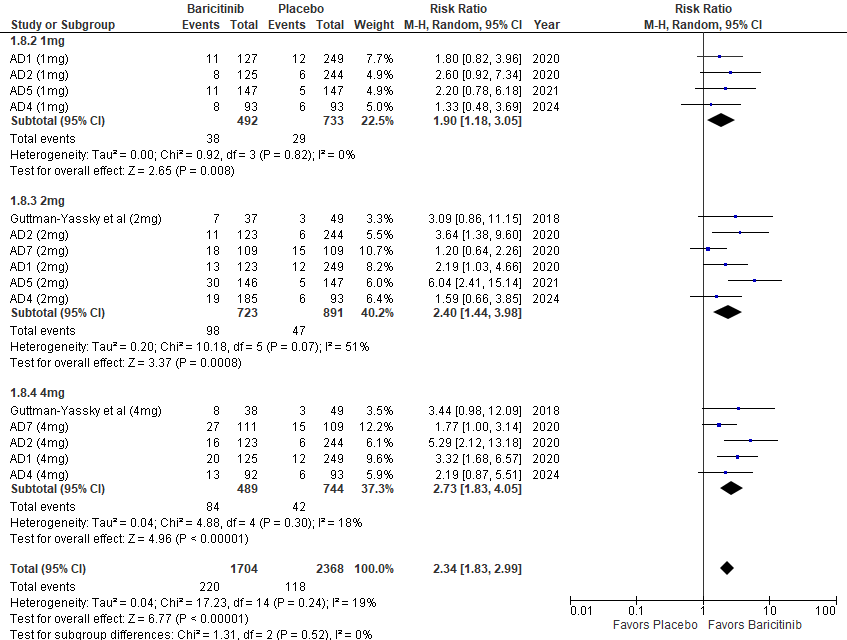


**Supplementary Figure 8: Forest plot for subgroup analysis of EASI 90 with/without TCS**

**
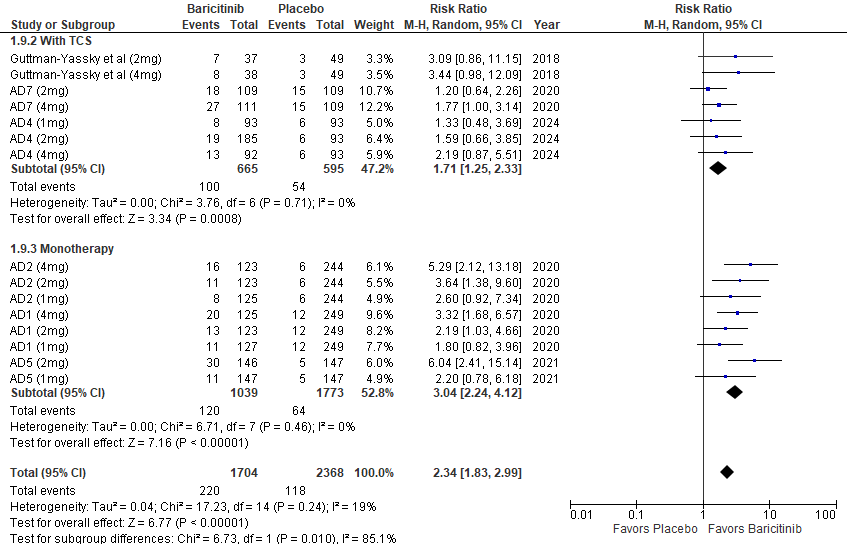
**

**Supplementary Figure 9: Forest plot for subgroup analysis of change from baseline in DLQI score**

**by dosage**

**
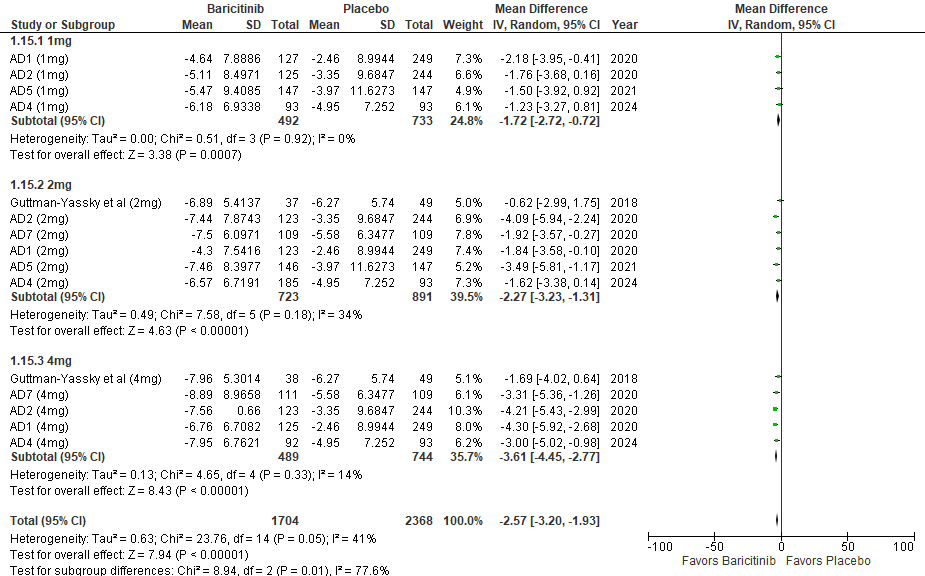
**

**Supplementary Figure 10: Forest plot for subgroup analysis of change from baseline in DLQI score with/without TCS**

**
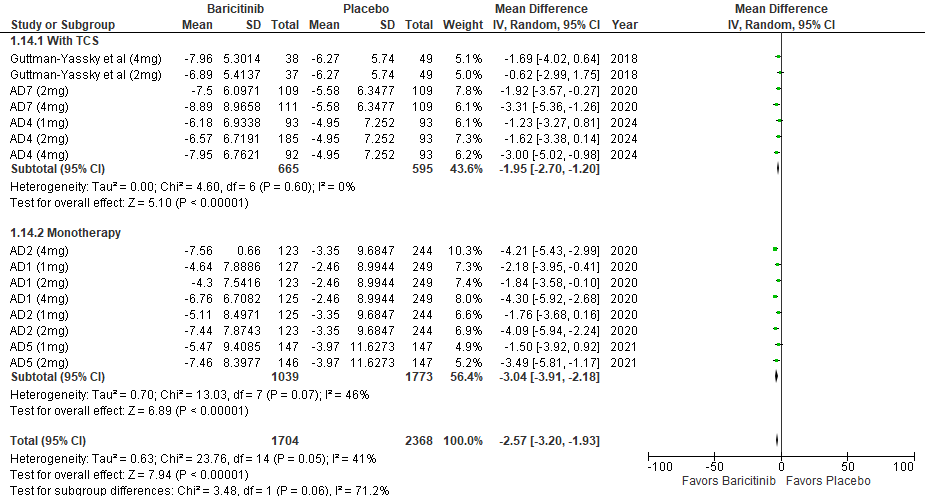
**

**Supplementary Figure 11: Forest plot for subgroup analysis of SCORAD 75 by dosage**

**
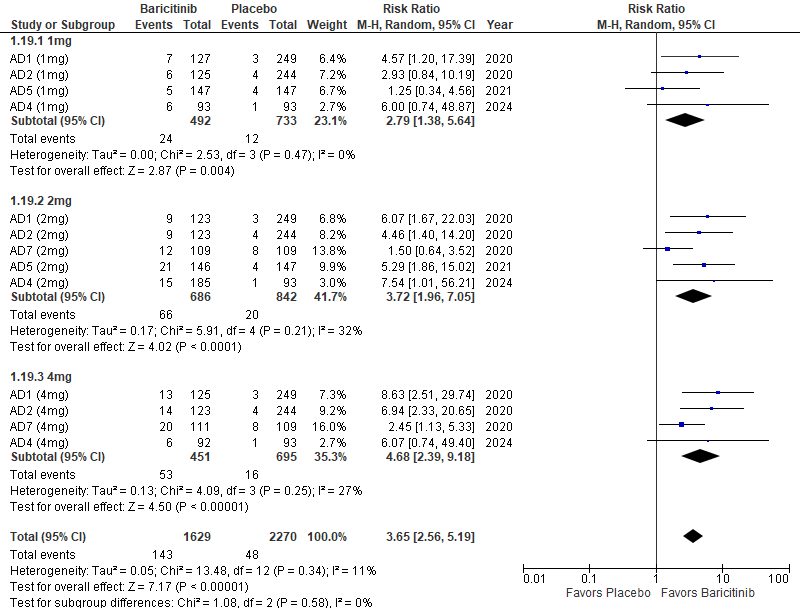
**

**Supplementary Figure 12: Forest plot for subgroup analysis of SCORAD 75 with/without TCS**

**
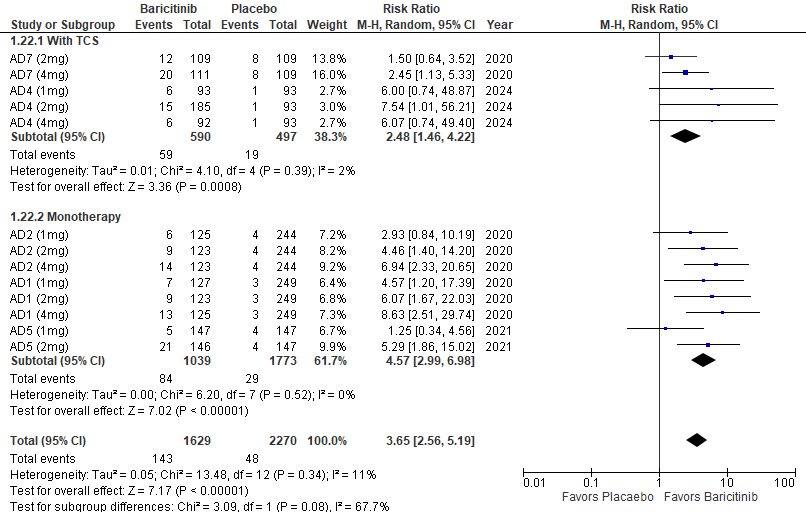
**

**Supplementary Figure 13: Forest plot for subgroup analysis of SCORAD 90 by dosage**

**
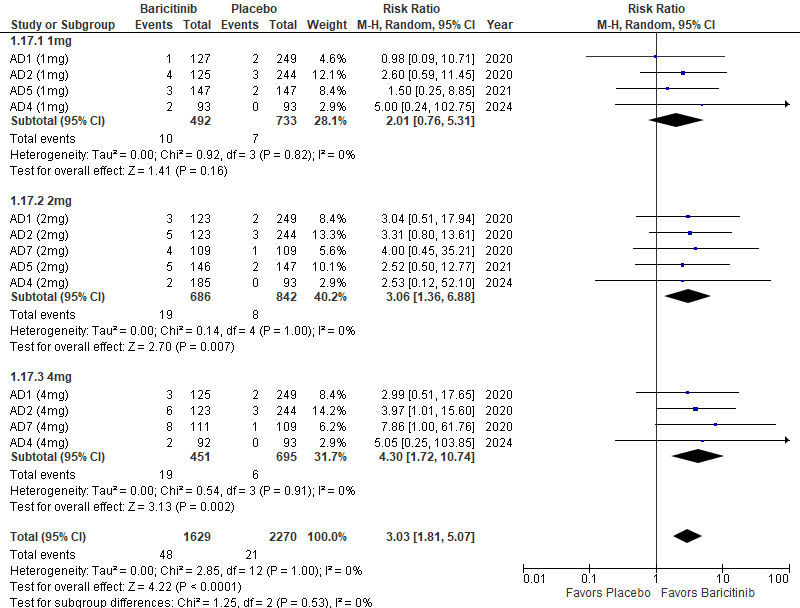
**

**Supplementary Figure 14: Forest plot for subgroup analysis of SCORAD 90 with/without TCS**

**
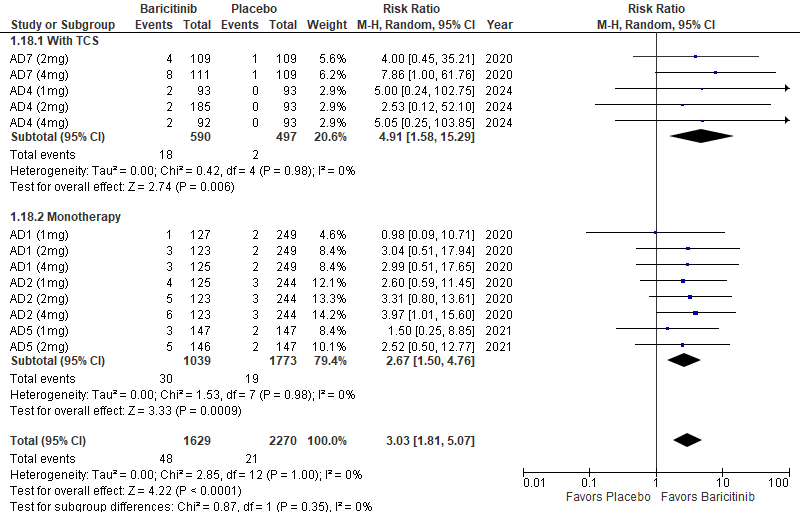
**

**Supplementary Figure 15: Forest plot for subgroup analysis of Itch NRS by dosage**

**
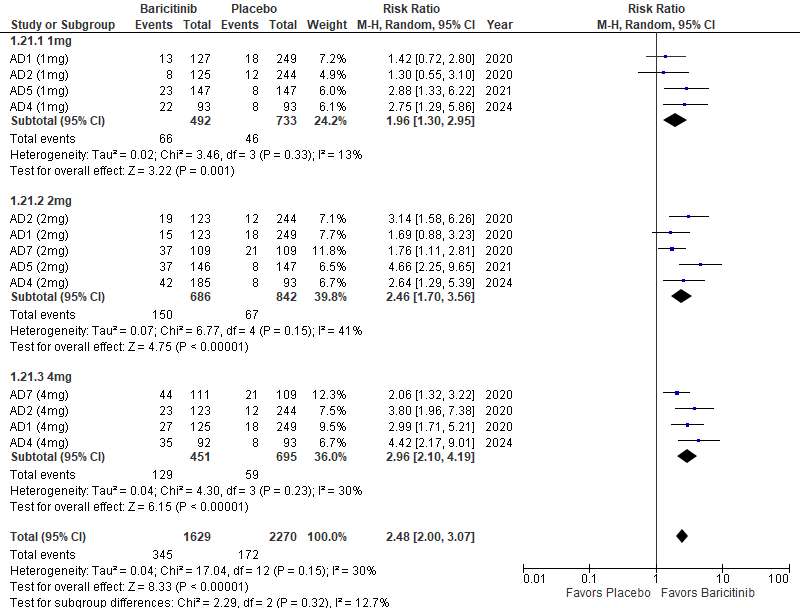
**

**Supplementary Figure 16: Forest plot for subgroup analysis of Itch NRS with/without TCS**

**
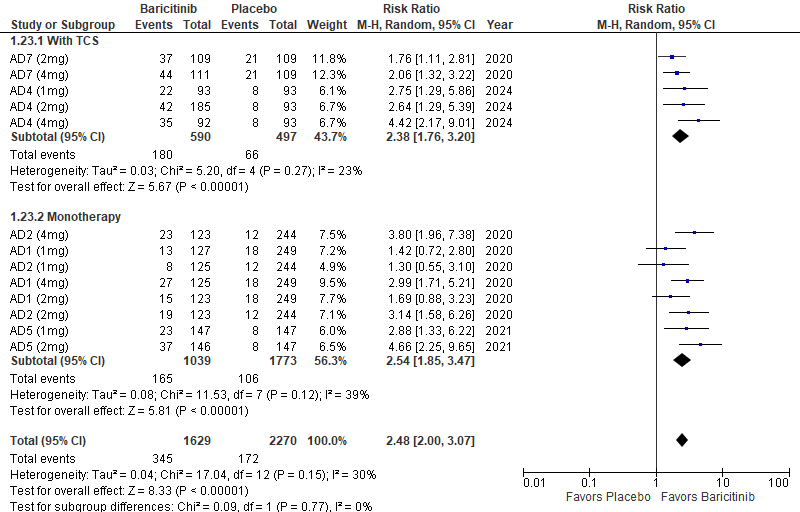
**

**Supplementary Figure 17: Forest plot for subgroup analysis of Skin infections requiring antibiotic treatment**

**by dosage**

**
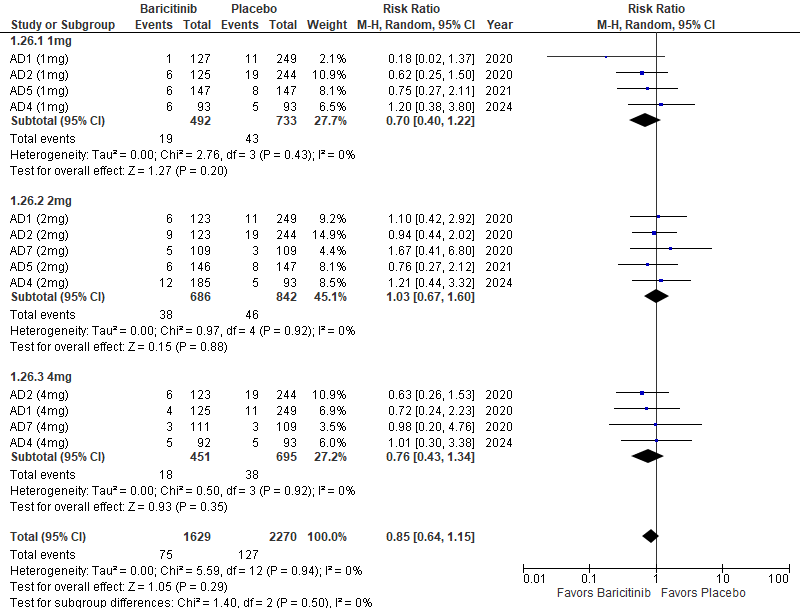
**

**Supplementary Figure 18: Forest plot for subgroup analysis of Skin infections requiring antibiotic treatment**

**with/without TCS**

**
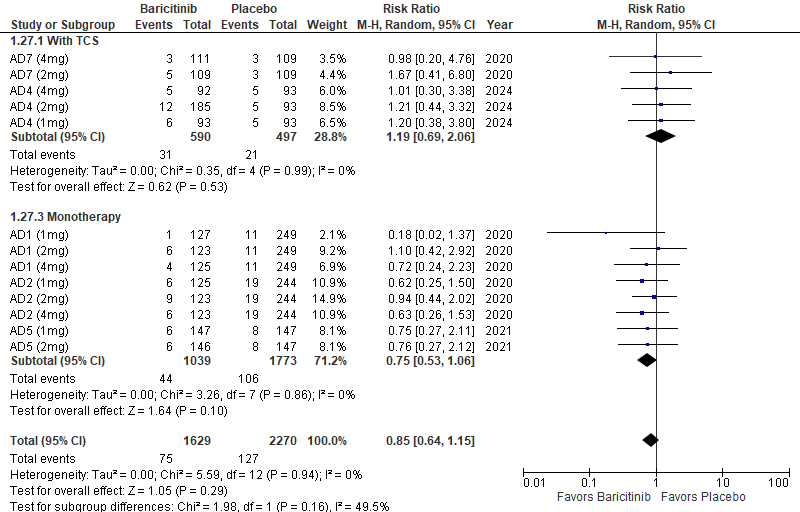
**

**Supplementary Figure 19: Forest plot for subgroup analysis of Skin Pain NRS by dosage**

**
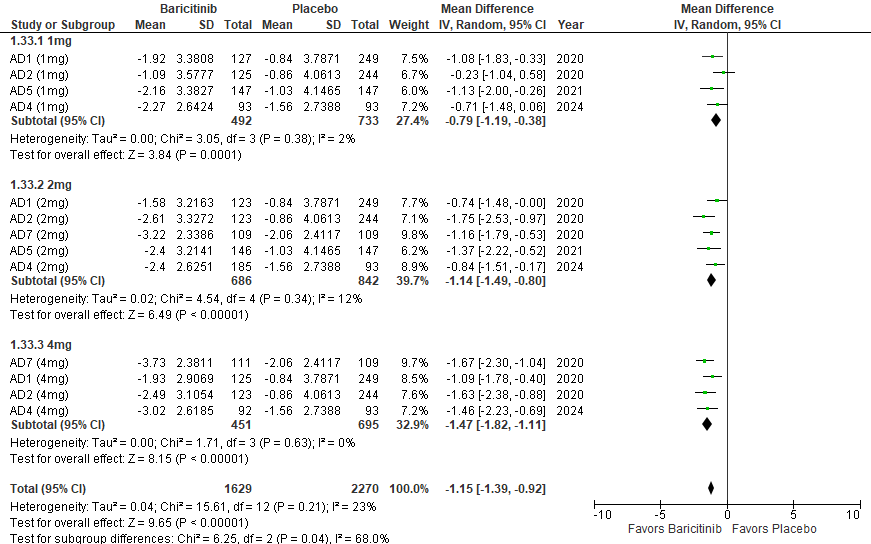
**

**Supplementary Figure 20: Forest plot for subgroup analysis of Skin Pain NRS with/without TCS**

**
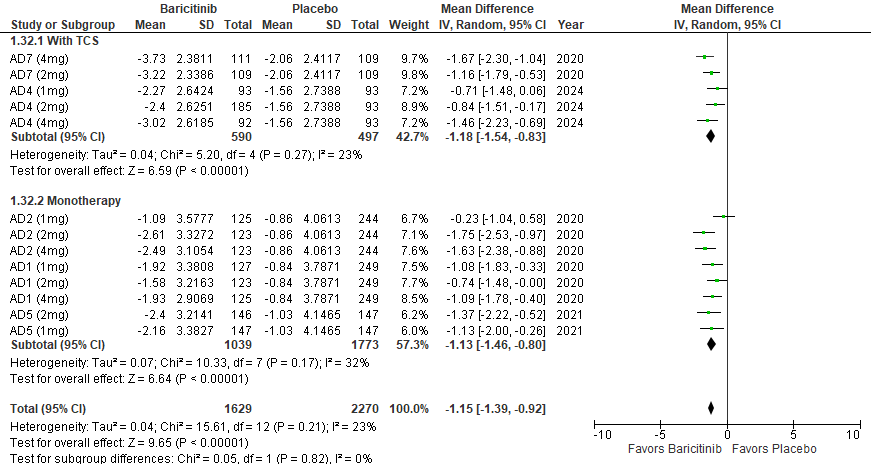
**

**Supplementary Figure 21: Forest plot for subgroup analysis of BSA Affected by dosage**

**
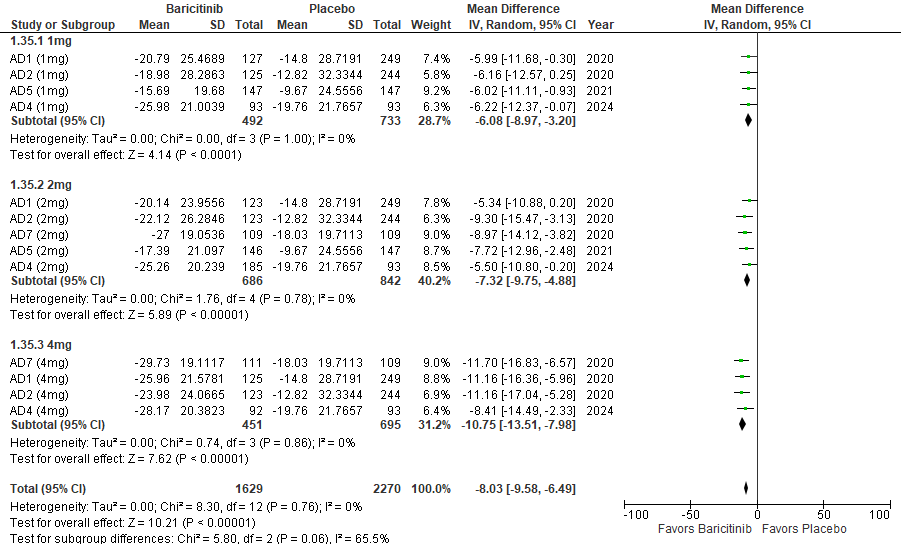
**

**Supplementary Figure 22: Forest plot for subgroup analysis of BSA Affected with/without TCS**

**
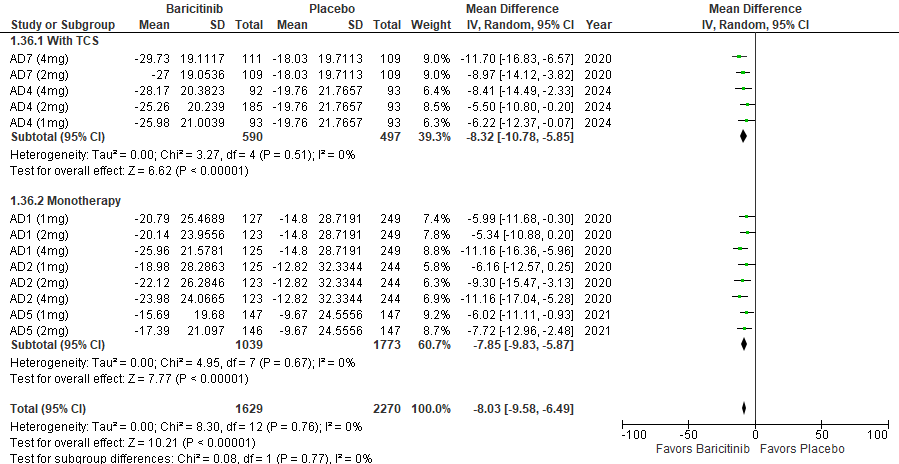
**

**Supplementary Figure 23: Forest plot for subgroup analysis of POEM by dosage**

**
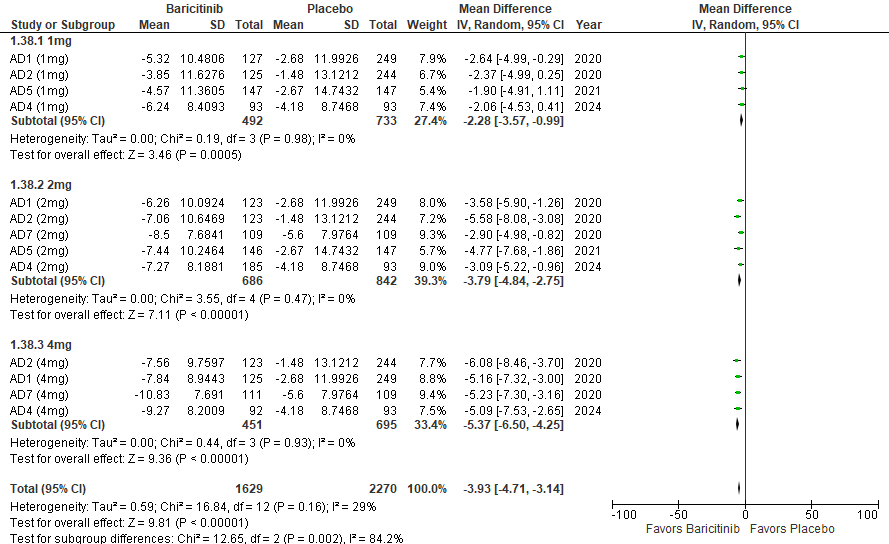
**

**Supplementary Figure 24: Forest plot for subgroup analysis of POEM with/without TCS**

**
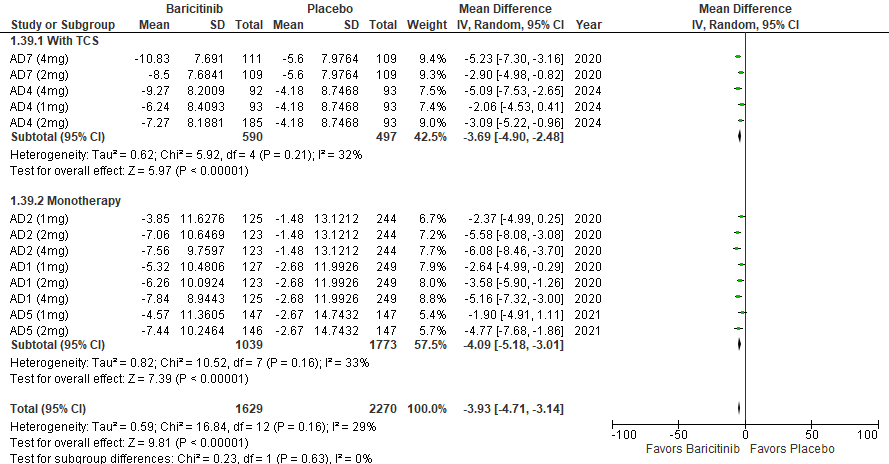
**

**Supplementary Figure 25: Forest plot for subgroup analysis of TEAEs by dosage**

**
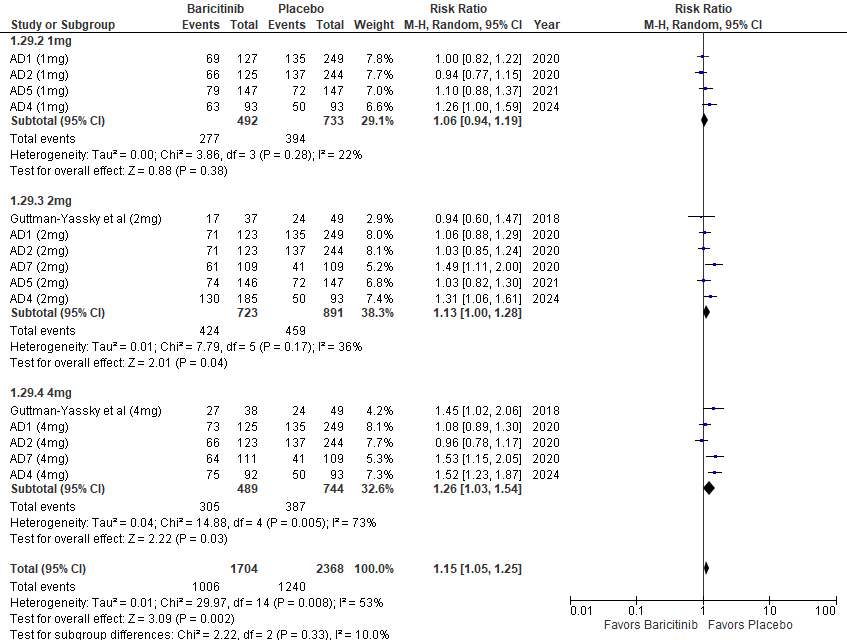
**

**Supplementary Figure 26: Forest plot for subgroup analysis of TEAEs with/without TCS**

**
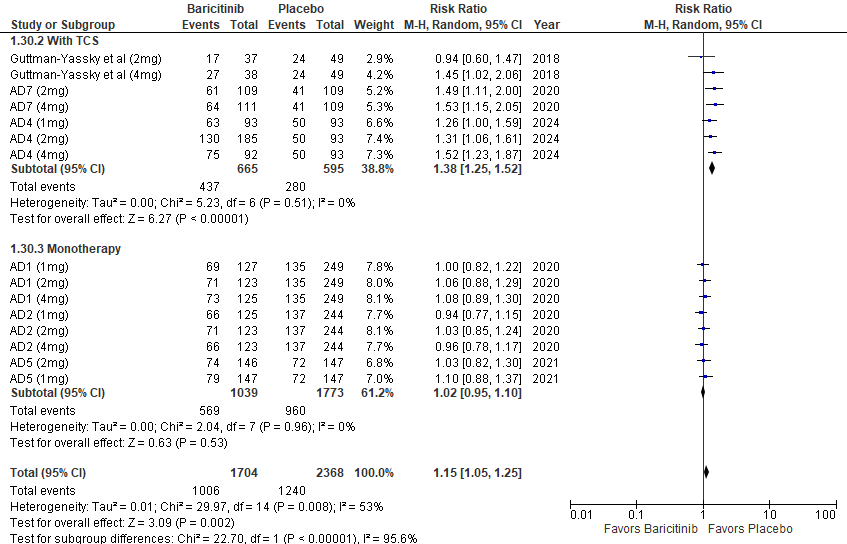
**
